# Time-Trends in Air Pollution Impact on Health in Italy, 1990–2019: An Analysis From the Global Burden of Disease Study 2019

**DOI:** 10.3389/ijph.2023.1605959

**Published:** 2023-06-02

**Authors:** Sara Conti, Carla Fornari, Pietro Ferrara, Ippazio C. Antonazzo, Fabiana Madotto, Eugenio Traini, Miriam Levi, Achille Cernigliaro, Benedetta Armocida, Nicola L. Bragazzi, Ennio Cadum, Michele Carugno, Giacomo Crotti, Silvia Deandrea, Paolo A. Cortesi, Davide Guido, Ivo Iavicoli, Sergio Iavicoli, Carlo La Vecchia, Paolo Lauriola, Paola Michelozzi, Salvatore Scondotto, Massimo Stafoggia, Francesco S. Violante, Cristiana Abbafati, Luciana Albano, Francesco Barone-Adesi, Antonio Biondi, Cristina Bosetti, Danilo Buonsenso, Giulia Carreras, Giulio Castelpietra, Alberico Catapano, Maria S. Cattaruzza, Barbara Corso, Giovanni Damiani, Francesco Esposito, Silvano Gallus, Davide Golinelli, Simon I. Hay, Gaetano Isola, Caterina Ledda, Stefania Mondello, Paolo Pedersini, Umberto Pensato, Norberto Perico, Giuseppe Remuzzi, Francesco Sanmarchi, Rocco Santoro, Biagio Simonetti, Brigid Unim, Marco Vacante, Massimiliano Veroux, Jorge H. Villafañe, Lorenzo Monasta, Lorenzo G. Mantovani

**Affiliations:** ^1^ Research Center on Public Health, University of Milan Bicocca, Monza, Italy; ^2^ Laboratory of Public Health, Auxologico Research Hospital—IRCCS, Milan, Italy; ^3^ Dipartimento di Anestesia, Rianimazione ed Emergenza Urgenza, Fondazione IRCCS Ca’ Granda Ospedale Maggiore Policlinico, Milano, Italy; ^4^ Institute for Risk Assessment Sciences (IRAS), Utrecht University, Utrecht, Netherlands; ^5^ Epidemiology Unit, Department of Prevention, Central Tuscany Local Health Authority, Florence, Italy; ^6^ Health Activities and Epidemiological Observatory Department, Health Authority Sicily Region, Parlemo, Italy; ^7^ Clinical Pathology Complex Hospital Unit, Health Authority Trapani Province, Trapani, Italy; ^8^ Department of Cardiovascular, Endocrine-Metabolic Disease and Aging, National Institute of Health (ISS), Rome, Italy; ^9^ Laboratory for Industrial and Applied Mathematics (LIAM), Department of Mathematics and Statistics, Faculty of Science, York University, Toronto, ON, Canada; ^10^ Department of Hygiene and Health Prevention and Complex Operative Unit Environmental Health and Innovative Projects, Health Protection Agency, Pavia, Italy; ^11^ Department of Clinical Sciences and Community Health, University of Milan, Milan, Italy; ^12^ Epidemiology Unit, Fondazione IRCCS Ca’ Granda Ospedale Maggiore Policlinico, Milan, Italy; ^13^ Servizio Epidemiologico Aziendale, Agenzia di Tutela della Salute di Bergamo, Bergamo, Italy; ^14^ Neurology, Public Health and Disability Unit, Carlo Besta Neurological Institute, Fondazione IRCCS Istituto Neurologico Carlo Besta, Milan, Italy; ^15^ Department of Public Health, University of Naples Federico II, Naples, Italy; ^16^ Department of Occupational and Environmental Medicine Epidemiology and Hygiene, Italian National Workers Compensation Authority (IIL), Monteporzio Catone, Italy; ^17^ International Network of Public Health and Environment Tracking (INPHET), Milan, Italy; ^18^ Department of Epidemiology, Lazio Region Health Authority (ASL RM1), Rome, Italy; ^19^ Occupational Health Unit, Department of Medical and Surgical Sciences, University of Bologna, Bologna, Italy; ^20^ Department of Juridical and Economic Studies, Faculty of Law, Sapienza University of Rome, Rome, Italy; ^21^ Department of Experimental Medicine, University of Campania Luigi Vanvitelli, Naples, Italy; ^22^ Department of Translational Medicine, University of Eastern Piedmont, Novara, Italy; ^23^ Department of General Surgery and Medical-Surgical Specialties, University of Catania, Catania, Italy; ^24^ Department of Oncology, Mario Negri Pharmacological Research Institute (IRCCS), Milano, Italy; ^25^ Department of Woman and Child Health and Public Health, Fondazione Policlinico Universitario A. Gemelli IRCCS (Agostino Gemelli University Polyclinic IRCCS), Rome, Italy; ^26^ Global Health Research Institute, Università Cattolica del Sacro Cuore (Catholic University of Sacred Heart), Rome, Italy; ^27^ Institute for Cancer Research, Prevention and Clinical Network (ISPRO), Florence, Italy; ^28^ Department of Medicine, University of Udine, Udine, Italy; ^29^ Department of Mental Health, Healthcare Agency “Friuli Occidentale”, Pordenone, Italy; ^30^ Department of Pharmacological and Biomolecular Sciences, University of Milan, Milan, Italy; ^31^ MultiMedica, IRCCS, Sesto San Giovanni, Italy; ^32^ Department of Public Health and Infectious Diseases, Sapienza University of Rome, Rome, Italy; ^33^ Institute of Neuroscience, National Research Council (CNR), Pisa, Italy; ^34^ IRCCS Istituto Ortopedico Galeazzi (Galeazzi Orthopedic Institute IRCCS), University of Milan, Milan, Italy; ^35^ Department of Dermatology, Case Western Reserve University, Cleveland, OH, United States; ^36^ Department of Biomedical and Neuromotor Sciences, University of Bologna, Bologna, Italy; ^37^ Department of Environmental Health Sciences, Mario Negri Institute for Pharmacological Research, Milan, Italy; ^38^ Institute for Health Metrics and Evaluation, University of Washington, Seattle, WA, United States; ^39^ Department of Health Metrics Sciences, School of Medicine, University of Washington, Seattle, WA, United States; ^40^ Department of Clinical and Experimental Medicine, University of Catania, Catania, Italy; ^41^ Department of Biomedical and Dental Sciences and Morphofunctional Imaging, Messina University, Messina, Italy; ^42^ Clinical Research Department, IRCCS Fondazione Don Carlo Gnocchi, Milan, Italy; ^43^ Department of Neurology, IRCCS Humanitas Research Hospital, Milan, Italy; ^44^ Istituto di Ricerche Farmacologiche Mario Negri IRCCS, Bergamo, Italy; ^45^ Daccude, Todi, Italy; ^46^ Department of Law, Economics, Management and Quantitative Methods, University of Sannio, Benevento, Italy; ^47^ WSB University in Gdańsk, Gdańsk, Poland; ^48^ Department of Medical, Surgical Sciences and Advanced Technologies, University of Catania, Catania, Italy; ^49^ Clinical Epidemiology and Public Health Research Unit, Burlo Garofolo Institute for Maternal and Child Health, Trieste, Italy

**Keywords:** air pollution, particulate matter, ozone, global burden of disease, air quality regulations

## Abstract

**Objectives:** We explored temporal variations in disease burden of ambient particulate matter 2.5 μm or less in diameter (PM_2.5_) and ozone in Italy using estimates from the Global Burden of Disease Study 2019.

**Methods:** We compared temporal changes and percent variations (95% Uncertainty Intervals [95% UI]) in rates of disability adjusted life years (DALYs), years of life lost, years lived with disability and mortality from 1990 to 2019, and variations in pollutant-attributable burden with those in the overall burden of each PM_2.5_- and ozone-related disease.

**Results:** In 2019, 467,000 DALYs (95% UI: 371,000, 570,000) were attributable to PM_2.5_ and 39,600 (95% UI: 18,300, 61,500) to ozone. The crude DALY rate attributable to PM_2.5_ decreased by 47.9% (95% UI: 10.3, 65.4) from 1990 to 2019. For ozone, it declined by 37.0% (95% UI: 28.9, 44.5) during 1990–2010, but it increased by 44.8% (95% UI: 35.5, 56.3) during 2010–2019. Age-standardized rates declined more than crude ones.

**Conclusion:** In Italy, the burden of ambient PM_2.5_ (but not of ozone) significantly decreased, even in concurrence with population ageing. Results suggest a positive impact of air quality regulations, fostering further regulatory efforts.

## Introduction

Air pollution represents a paradigm of risk factor associated to relatively modest increases in the individual risk, but to substantial disease burden at the population level [1]. The World Health Organization (WHO) recognized air pollution as a health risk factor in 1958 [2], with effects ranging from subclinical lesions to premature death [3, 4]. Although directives have been thereafter issued, it remains a substantial public health concern owed to its mortality and disability burden [5–7]. In 2008, the European Union (EU) introduced the “Air Quality Directive”, fixing target values for long-term concentrations of air pollutants [4]. As a plausible effect, a reduction of 22% in annual mean concentrations of PM_2.5_ was observed across Europe from 2009 to 2018. Conversely, an increase in ozone concentrations was registered over the same period, owed to increasing in precursors emission and changes in regional climate characteristics [1, 8]. The 2021 WHO Global Air Quality Guidelines updated air quality levels, focusing on six pollutants: particulate matter with diameter equal or smaller than 2.5 and 10 µm (PM_2.5_ and PM_10_), ozone, nitrogen dioxide, sulfur dioxide and carbon monoxide [3]. In this frame, it is compelling to understand the health impact of regulation-driven modifications in pollutant concentrations.

The Global Burden of Diseases (GBD) Study 2019 graded air pollution as fourth-ranking risk factor for mortality and disability-adjusted life years (DALYs) globally, accounting for 85.6 deaths (95% Uncertainty Interval [95% UI]: 75.7, 96.1) and 2791.1 DALYs (95% UI: 2468.8, 3141.4) per 100,000 people [7]. These estimates quantify health impacts attributable to exposure to ambient and household PM_2.5_ and ambient ozone, and account for morbidity and mortality of selected diseases associated with pollution: chronic obstructive pulmonary disease (COPD), lower respiratory infections (LRI), ischemic heart disease (IHD), stroke, tracheal bronchus and lung (TBL) cancer, type 2 diabetes mellitus (T2DM), premature birth and decreased birthweight [1, 7].

Specific evidences from the GBD Study underlined that deaths and DALYs due to PM_2.5_ long-term exposure globally increased from 1990 to 2015. Meanwhile, an increment of ozone-attributable COPD deaths was observed [9]. These trends might result from several phenomena that add up to the variation in pollutants concentrations, such as changes in the population age structure or variations in mortality and morbidity rates [8–13].

Using GBD estimates, we investigated temporal variations in disease burden from long-term exposure to ambient PM_2.5_ and ozone from 1990 to 2019 in Italy, with the aim of disentangling the effect of the reduction in air pollution concentration from demographic (population ageing) and epidemiologic (mortality and morbidity rates) population dynamics.

## Methods

### Overview

The GBD Study provides comprehensive global estimates of disease burden, such as incidence, prevalence, mortality, years of life lost (YLLs), years lived with disability (YLDs), healthy life expectancy (HALE) and DALYs, for 204 countries and territories [7]. It also applies a comprehensive and standardized method to identify risk factors and risk-outcome associations by disease cause. Risk factors are organized in five hierarchical nested levels; risk-outcome pairs are assessed for inclusion based on availability and strength of the evidence for a causal association, and on the feasibility of developing complete estimates of exposure levels [7, 14].

The burden attributable to a risk factor is estimated for each risk–outcome pair based on the overall estimate of the outcome burden, spatial and temporal exposure estimates for the risk factor, the theoretical minimum risk exposure level (TMREL) and the relative risk, or dose-response function, describing the association between risk factor and outcome [15].

Here, we focus on the burden of long-term exposure to ambient PM_2.5_ (GBD level four risk factor) and ozone (GBD level three risk factor) pollution. Detailed descriptions of data sources, metrics and methods are available elsewhere [7]. The study is compliant with the Guidelines for Accurate and Transparent Health Estimates Reporting [16].

### Ambient PM_2.5_ Exposure and Associated Risk-Outcome Pairs

Input data used by GBD 2019 to estimate population-weighted exposure to ambient PM_2.5_ included ground measurements, satellite-based estimates, chemical transport model simulations, and population estimates. GBD 2019 data sources for ground measurements of PM_2.5_ consisted of updated measurements from GBD 2017 and additional measurements provided by the WHO Global Ambient Air Quality Database in 2018. A hierarchy of conversion factors (PM_2.5_/PM_10_ ratios) was used to obtain PM_2.5_ values for locations containing only measurements of PM_10_. The satellite-based estimates combined information on aerosol optical depth retrievals from multiple satellites, chemical transport model simulations and information on land use, and were available on a spatial resolution of 0.1^0^ × 0.1^0^ (which corresponds to 11 × 11 km at the equator) [17]. Population estimates were derived from the Gridded Population of the World database for the years 1990, 1995 (3rd version), and 2000, 2005, 2010, 2015, 2020 (4th version), and natural spline interpolation was used to calculate population estimates in intermediate years. PM_2.5_ estimates in Italy were calculated using a Bayesian hierarchical calibration model, thus combining information from satellite retrievals, chemical transport model simulations and population estimates calibrated with ground monitor PM_2.5_ estimates. The TMREL for ambient particulate matter was estimated on a uniform distribution with lower and upper bounds corresponding to 2.4 and 5.9 μg/m^3^ based on the average of the minimum and 5^th^ percentiles of exposure distributions to air pollution in the cohort studies used to produce the GBD estimates.

Risk-attributable disease burden for ambient PM_2.5_ was computed for risk-outcome pairs validated for inclusion using the scientific literature. Such validation procedure identified the following GBD level three outcomes (causes): IHD, stroke, COPD, T2DM, LRI, TBL cancer, and neonatal disorders [7, 14].

In order to ascertain the shape of the dose-response relationship for each outcome, the GBD 2019 adopted the Meta-Regression-Bayesian Regularized Trimmed (MR-BRT) strategy, with input data from studies assessing the effect of PM_2.5_ ambient and household pollution [6, 16, 18–20].

### Ambient Ozone Exposure and Associated Risk-Outcome Pairs

GBD 2019 definition of ozone ambient air pollution is the highest seasonal (6 months) average of 8-h daily maximum ozone concentrations measured as parts per billion (ppb), for each 0.1^0^ × 0.1^0^ grid cell on a global scale. Ozone ground measurements obtained from the Tropospheric Ozone Assessment Report and continent-specific chemical transport models provided by the Chemistry-Climate Model Initiative were combined in a single geo-statistical modelling tool, the Bayesian Maximum Entropy, to estimate the exposure to ambient ozone pollution globally for the period 1990–2017. Subsequently, a log-linear model was run to extrapolate ambient ozone pollution exposure for the years 2018 and 2019. The TMREL for ambient ozone air pollution ranged between 29.1 and 35.7 ppb [7, 10].

In GBD Study 2019, estimation of ambient ozone pollution attributable disease burden is based on a literature review exploring long-term ozone exposure and COPD mortality including five cohort studies, followed by MR-BRT risk splines estimation [7, 14].

### Analysis

We used GBD estimates for population-weighted exposure, population age structure, deaths, DALYs, YLLs and YLDs attributable to ambient PM_2.5_ and ozone in Italy from 1990 to 2019 [21, 22]. We also obtained GBD estimate for the global population age structure in 2019. For ozone, estimated DALYs corresponded to YLLs, as YLDs were always null, since literature only supported the association with COPD mortality. We downloaded the same measures also for the overall burden of each disease associated with PM_2.5_ and ozone. Crude and age-standardized rates per 100,000 person-years with 95% UI were considered for all estimates. Age-standardized rates were computed through a direct standardization, using GBD 2019 World Standard Population as a reference [23].

The temporal evolution of the burden attributed to ambient PM_2.5_ and ozone pollution from 1990 to 2019 was analyzed both as annual rates and percent variation (95% UI) in rates from 1990 to 2019, 1990 to 2010, 2010 to 2019. This analysis was stratified by age, sex and cause-specific disease burden.

To highlight the contribution of demographic and epidemiologic dynamics in the variation of disease burden attributable to ambient pollution, we first compared time changes of crude and age-standardized rates attributable to ambient PM_2.5_ and ozone pollution, as the discrepancy in the temporal evolution of these two measures is related to population aging. Crude rates depict the real estimated variation based on the age-structure of the Italian population, while age-standardized ones report the expected variation if the population maintained a fixed age-structure equal to that of the global one. Therefore, the decrease observed for age-standardized rates can be interpreted as the expected decrease in the absence of population ageing. Then, we compared time changes in overall diseases burden to that attributable to PM_2.5_ and ozone, through age-standardized rates. These comparisons were carried out both graphically and on percent variations (95% UI) over 1990–2010 and 2010–2019 periods.

## Results

### The Burden of Ambient Air Pollution in 2019

In 2019, in Italy, ambient PM_2.5_ pollution accounted for 24,700 deaths (95% UI: 19,200, 30,000) and 467,000 DALYs (95% UI: 371,000, 570,000), corresponding to 3.8% (95% UI: 3.0, 4.7) of total deaths, and 2.6% (95% UI: 2.0, 3.2) of total DALYs. The burden attributable to ambient ozone pollution was lower, with 3,490 deaths (95% UI: 1,600, 5,390) and 39,600 DALYs (95% UI: 18,300, 61,500), corresponding to 0.5% (95% UI: 0.3, 0.8) and 0.2% (95% UI: 0.1, 0.3), respectively.

Crude DALY rates per 100,000 inhabitants due to ambient PM_2.5_ amounted to 773.5 (95% UI: 614.5, 945.0). The burden was higher among males (924.5, 95% UI: 734.2, 1122.2) than in females (630.5, 95% UI: 492.3, 781.1). Children aged up to 5 years had higher DALY rates (149.1, 95% UI: 99.9, 206.6) than those aged 5 to 14 (0.9, 95% UI: 0.5, 1.4). Within the following age classes, an increasing trend emerged, reaching a maximum of 1944.8 (95% UI: 1511.9, 2391.0) among people aged 75 or more. The top three conditions associated to PM_2.5_ exposure in relation to DALY rate per 100,000 were IHD, T2DM and stroke (Table 1, Supplementary Tables S1–S3).

**TABLE 1:** Crude and age-standardized rates of disability-adjusted life years (DALYs) due to ambient particulate matter and ozone pollution in 2019. Temporal variation from 1990 to 2010, 2010 to 2019 and from 1990 to 2019 (Global Burden of Disease Study, Italy, 1990–2019).

	Crude DALY rate per 100,000 inhabitants				Age-standardized DALY rate per 100,000 inhabitants			
	Estimate (95% UI) for 2019	Percent variation (95% UI) 1990–2010	Percent variation (95% UI) 2010–2019	Percent variation (95% UI) 1990–2019	Estimate (95% UI) for 2019	Percent variation (95% UI) 1990–2010	Percent variation (95% UI) 2010–2019	Percent variation (95% UI) 1990–2019
Ambient PM_2.5_ pollution								
Total	773.51 (614.49, 944.96)	−35.4 (−56.1, 10.6)	−19.4 (−23.8, −15.8)	−47.9 (−65.4, −10.3)	357.49 (284.57, 435.66)	−51.0 (−66.2, −18.2)	−28.7 (−32.9, −25.4)	−65.1 (−76.4,−41.9)
Sex								
Males	924.49 (734.23, 1122.18)	−37.8 (−57.5, 6.0)	−21.3 (−25.6, −17.6)	−51.0 (−67.3, −16.0)	473.69 (379.03, 574.07)	−52.3 (−67.4, −20.4)	−31.4 (−35.4, −28.0)	−67.3 (−77.8, −45.2)
Females	630.48 (492.28, 781.10)	−31.6 (−53.7, 18.2)	−16.7 (−21.4, −12.7)	−43.0 (−62.2, −1.7)	257.11 (203.30, −315.61)	−49.3 (−65.1, −15.0)	−25.3 (−30.5, −20.9)	−62.1 (−74.3, −37.4)
Age class								
Under 5	149.06 (99.88, 206.60)	−65.1 (−77.2, −47.6)	−38.6 (−61.3, −4.7)	−78.5 (−86.9, −66.9)				
5–14	0.88 (0.53, 1.37)	−71.1 (−83.3, −41.9)	−37.3 (−43.4, −30.4)	−81.9 (−89.6, −63.4)				
15–49	130.68 (101.15, 162.50)	−41.2 (−59.0, −1.5)	−21.0 (−26.9, −15.6)	−53.5 (−68.2, −21.6)				
50–74	568.38 (443.46, 709.92)	−48.3 (−64.9, −10.0)	−30.9 (−34.9, −27.7)	−64.3 (−76.3, −38.6)				
75 plus	1944.78 (1511.85, 2391.02)	−47.2 (−65.1, −5.8)	−29.2 (−32.9, −26.1)	−62.6 (−75.7, −33.3)				
Cause								
Ischemic heart disease	212.66 (166.32, 263.14)	−48.3 (−64.7, −12.1)	−21.9 (−26.2, −18.1)	−59.6 (−73.0, −30.1)	96.18 (76.55, 118.07)	−59.9 (−72.6, −31.7)	−31.3 (−35.1, −28.1)	−72.5 (−81.4, −53.0)
Type 2 diabetes mellitus	173.82 (105.80, 259.13)	29.0 (−6.5, 103.0)	−13.2 (−20.0, −7.4)	11.9 (−21.8, 77.7)	79.15 (48.50, 117.64)	−0.2 (−27.8, 56.6)	−20.6 (−27.0, −15.0)	−20.8 (−44.9, 25.6)
Stroke	146.38 (116.55, 178.93)	−51.4 (−68.4, −12.4)	−21.4 (−25.8, −17.4)	−61.8 (−75.7, −31.9)	64.08 (51.43, 77.88)	−63.2 (−75.8, −33.7)	−30.4 (−34.3, −27.1)	−74.4 (−83.5, −54.4)
Tracheal, bronchus, and lung cancer	130.40 (92.42, 176.52)	−27.3 (−51.6, 24.7)	−22.4 (−27.8, −18.0)	−43.7 (−63.0, −1.1)	61.78 (43.77, 83.57)	−41.9 (−61.2, −0.5)	−30.7 (−35.5, −26.7)	−59.7 (−73.6, −29.8)
Chronic obstructive pulmonary disease	86.06 (57.28, 119.48)	−18.8 (−51.2, 58.3)	−14.8 (−20.1, −9.8)	−30.8 (−59.1, 34.0)	32.79 (22.13, 45.53)	−42.5 (−65.5, 11.6)	−27.7 (−32.2, −23.5)	−58.5 (−75.3, −19.9)
Lower respiratory infections	18.66 (11.06, 28.97)	−40.2 (−65.1, 16.8)	−10.7 (−16.7, −5.5)	−46.6 (−69.2, 5.4)	7.94 (4.77, 12.18)	−63.4 (−78.8, −28.2)	−28.7 (−33.3, −24.8)	−73.9 (−84.7, −49.3)
Neonatal disorders	5.46 (3.57, 7.65)	−63.4 (−77.1, −43.1)	−48.7 (−68.5, −18.2)	−81.2 (−88.6, −70.0)	15.36 (10.04, 21.54)	−61.4 (−75.8, −39.8)	−35.3 (−60.4, 3.1)	−75.0 (−84.8, −60.0)
Ambient ozone pollution^a^								
Total	65.63 (30.31, 101.92)	−37.0 (−44.5, −28.9)	44.8 (35.5, 56.3)	−8.8 (−18.6, 0.4)	23.56 (10.98, 36.79)	−56.8 (−61.8, −51.3)	22.1 (14.5, 31.7)	−47.3 (−52.3, −41.1)
Sex								
Males	83.77 (38.92, 129.07)	−44.6 (−50.9, −37.8)	42.9 (32.3, 55.7)	−20.9 (−28.6, −12.5)	36.02 (16.73, 55.31)	−61.1 (−65.5, −56.3)	15.8 (7.7, 26.3)	−55.0 (−59.1, −50.4)
Females	48.44 (22.56, 76.41)	−18.0 (−29.4, −6.0)	47.8 (35.8, 61.7)	21.2 (2.9, 37.5)	14.82 (6.82, 23.66)	−45.3 (−52.2, −36.1)	26.4 (17.8, 37.9)	−30.9 (−38.4, −20.6)
Age class								
15–49	2.30 (1.06, 3.57)	−45.4 (−53.8, −34.0)	32.0 (19.9, 47.9)	−27.9 (−37.4, −12.2)				
50–74	28.30 (13.22, 44.01)	−62.6 (−67.5, −55.7)	20.7 (11.3, 32.3)	−54.9 (−59.9, −47.1)				
75 plus	251.35 (114.29, 389.58)	−48.4 (−54.7, −42.7)	23.4 (15.1, 33.8)	−36.4 (−43.6, −30.1)				

^a^ The burden of ozone is limited to mortality for chronic obstructive pulmonary disease among people aged 15 and more. UI, uncertainty interval.

As for ambient ozone pollution, the total crude DALY rate per 100,000 inhabitants was 65.6 (95% UI: 30.3, 101.9), (Table 1). The burden was higher among males (83.8, 95% UI: 38.9, 129.1) than in females (48.4, 95% UI: 22.6, 76.4), and it increased with age, starting from 2.3 (95% UI: 1.1, 3.6) among people aged 15–49 and rising to 251.4 (95% UI: 114.3, 389.6) among those aged 75 or more (Table 1).

Age-standardized DALY rates were 357.5 (95% UI: 284.6, 435.7) and 23.6 (95% UI: 11.0, 36.8), respectively for ambient PM_2.5_ and ozone. The difference between males and females persisted: estimated DALY rates were respectively 473.7 (95% UI: 379.0, 574.1) and 257.1 (95% UI: 203.3, 315.6) for PM_2.5_, and 36.0 (95% UI: 16.7, 55.3) and 14.8 (95% UI: 6.8, 23.7) for ozone. Furthermore, when stratifying by condition associated with PM_2.5_, the pollutant burden confirmed to be highest for IHD and T2DM (Table 1).

A thorough description of mortality, YLLs and YLDs attributable to PM_2.5_ and ozone is reported in Supplementary Tables S1–S3. The overall crude mortality rate was 40.9 (95% UI: 31.8, 49.8) per 100,000 inhabitants, while the age-standardized one was 14.8 (95% UI: 11.8, 17.9) (Supplementary Table S1). YLLs outweighed YLDs, with age-standardized rates of respectively 269.8 (95% UI: 220.0, 320.5) and 87.7 (95% UI: 56.1, 125.4) (Supplementary Tables S2, S3). Differences between sexes were also confirmed for mortality and YLL rates. The distribution among age classes mirrored that observed for DALYs for all other burden measures. Notably, YLDs exceeded YLLs for T2DM, and they represented a consistent share of the total DALYs for COPD and stroke.

### Temporal Trends

**FIGURE 1:**
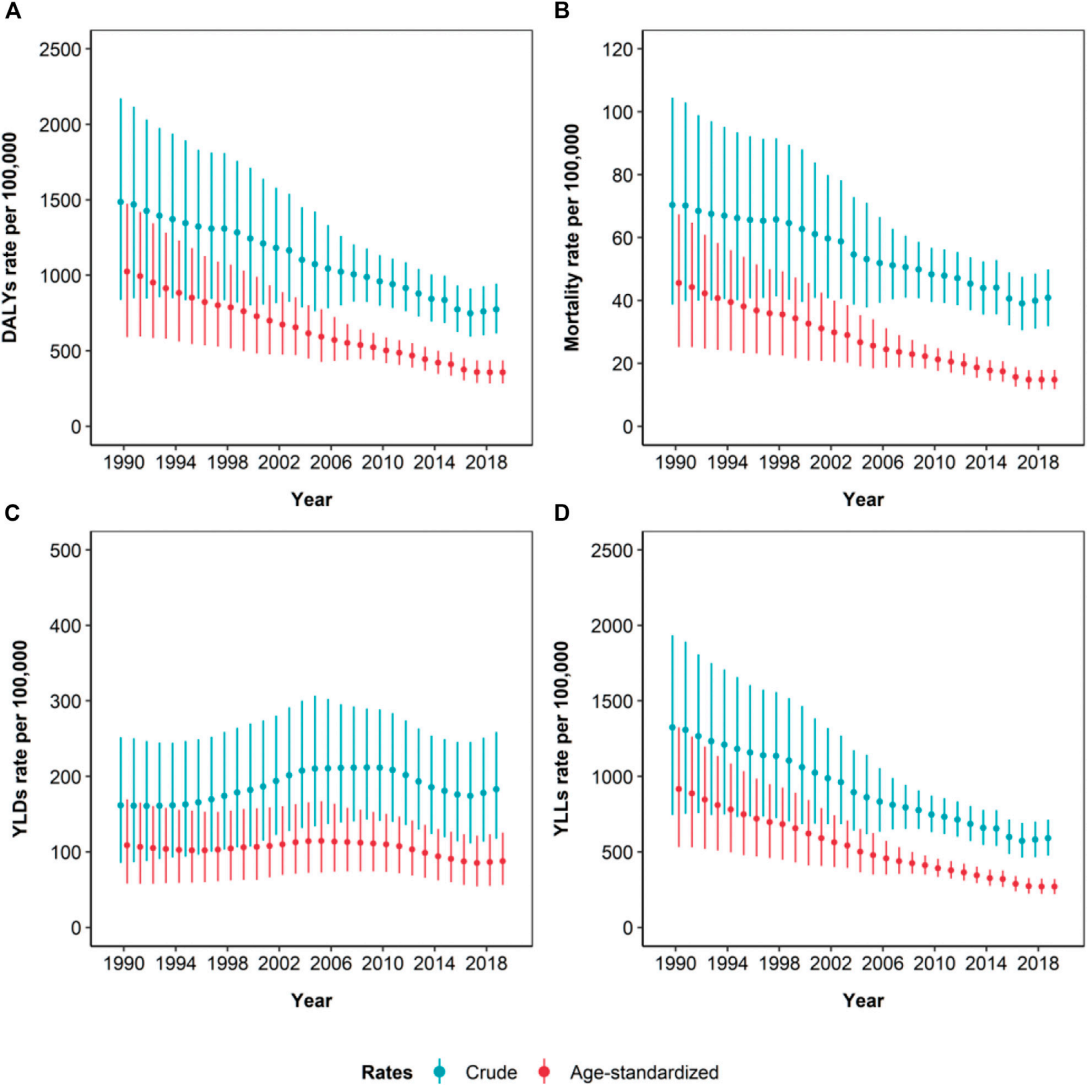
Time-series of the estimated crude and age-standardized rates (per 100,000 inhabitants) of disability adjusted life years (DALYs) **(A)**, mortality **(B)**, years lived in disability (YLDs) **(C)**, and years of life lost (YLLs) **(D)**, due to ambient particulate matter pollution, from 1990 to 2019. Whiskers represent 95% Uncertainty Intervals (Global Burden of Disease Study, Italy, 1990–2019).

**FIGURE 2:**
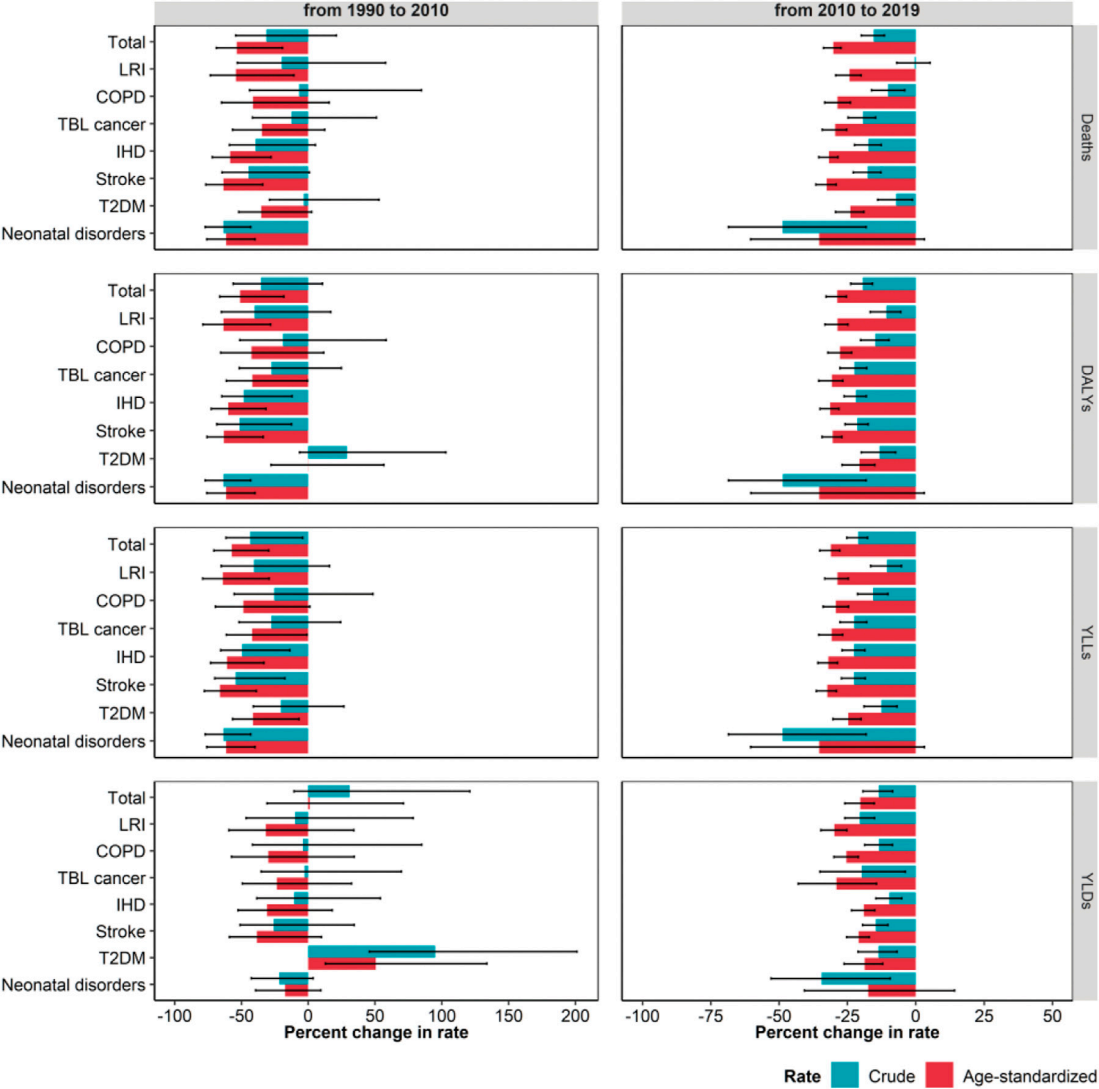
Estimated percent change in crude and age-standardized rates (per 100,000 inhabitants) of mortality (deaths), disability adjusted life years (DALYs), years of life lost (YLLs) and years lived in disability (YLDs) due to ambient particulate matter pollution, from 1990 to 2010 and from 2010 to 2019, stratified by cause. Whiskers represent 95% Uncertainty Intervals. LRI, Lower respiratory infections; COPD, Chronic obstructive pulmonary disease; TBL, Tracheal, bronchus and lung; IHD, Ischemic heart disease; T2DM, Type 2 diabetes mellitus (Global Burden of Disease Study, Italy, 1990–2019).

The population-weighted average concentration of PM_2.5_ decreased from 26.9 μg/m^3^ in 1990 to 16.1 μg/m^3^ in 2019 (Supplementary Figure S1). This was mirrored by a clear decreasing trend in the burden from 1990 to 2019 in terms of crude and age-standardized mortality, DALY and YLL rates, while YLD rates showed a rather stable trend (Figure 1). However, variations in crude rates were significantly lower than those observed for age-standardized rate. Crude DALY, mortality and YLL rates decreased by 47.9% (95% UI: 10.3, 65.4), 41.9% (95% UI: 3.2, 62.1) and 55.4% (95% UI: 22.6, 70.5), while the corresponding age-standardized rates decreased by 65.1% (95% UI: 41.9, 76.4), 67.5% (95% UI: 43.1, 78.8) and 70.5% (95% UI: 51.1, 80.1) (Table 1; Supplementary Tables S1–S3). When comparing the periods 1990–2010 and 2010–2019, significant differences in crude and age-standardized rates variations persisted only within the second period (Table 1; Figure 2). Focusing on DALYs, crude rates declined by 19.4% (95% UI: 15.8, 23.8), while the age-standardized ones decreased by 28.7% (95% UI: 25.4, 32.9). Variations observed for mortality and YLLs were similar to those of DALYs while YLDs showed slightly milder declines (Table 1; Supplementary Tables S1–S3; Figure 2). Focusing on age-specific rates, the decrease appeared to be slightly higher among people aged less than 14 for all the measures, while the minimum decrease in terms of DALYs was observed for people aged 15–49, due to a 29.5% (95% UI: 25.7, 33.6) decrease in YLL rates that is partially counterbalanced by a 6.8% (95% UI: −5.1, 18.3) increase in YLD rates (Table 1; Supplementary Tables S1–S3). Crude 2010–2019 DALY rates decrease ranged from 10.7% (95% UI: 5.5, 16.7) for LRI to 48.7% (95% UI: 18.2, 68.5) for neonatal disorders, but when considering age-standardized rates, all decreases were close to 30%, with the exception of T2DM (20.6%, 95% UI: 15.0, 27.0). A similar consideration applied to YLLs, while differences were even more pronounced when considering mortality (Figure 2 and Supplementary Tables S1–S3).

**FIGURE 3:**
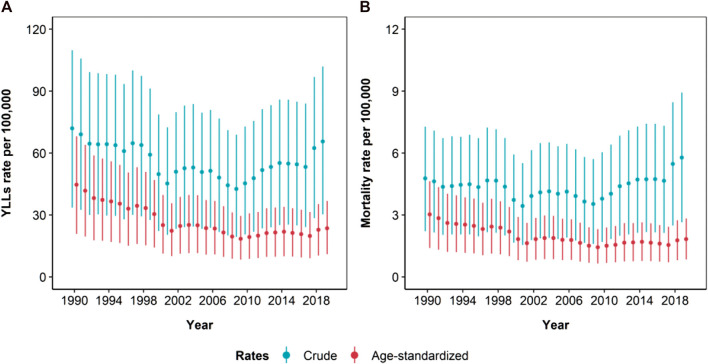
Time-series of the estimated crude and age-standardized rates (per 100,000 inhabitants) of years of life lost (YLLs) (A)* and mortality **(B)** due to ambient ozone pollution, from 1990 to 2019. Whiskers represent 95% Uncertainty Intervals (Global Burden of Disease Study, Italy, 1990–2019). *YLLs account for the whole DALYs amount.

Ambient ozone pollution burden described no clear decline during the whole period 1990–2019. Indeed, crude DALY and mortality rates declined by 37.0% (95% UI: 28.9, 44.5) and 20.6% (95% UI: 11.5, 30.4) during 1990–2010, but they increased by 44.8% (95% UI: 35.5, 56.3) and 52.6% (95% UI: 42.9, 64.8) during the following period. A similar trend was observed for age-standardized rates (Table 1; Supplementary Table S1; Figure 3).

**FIGURE 4:**
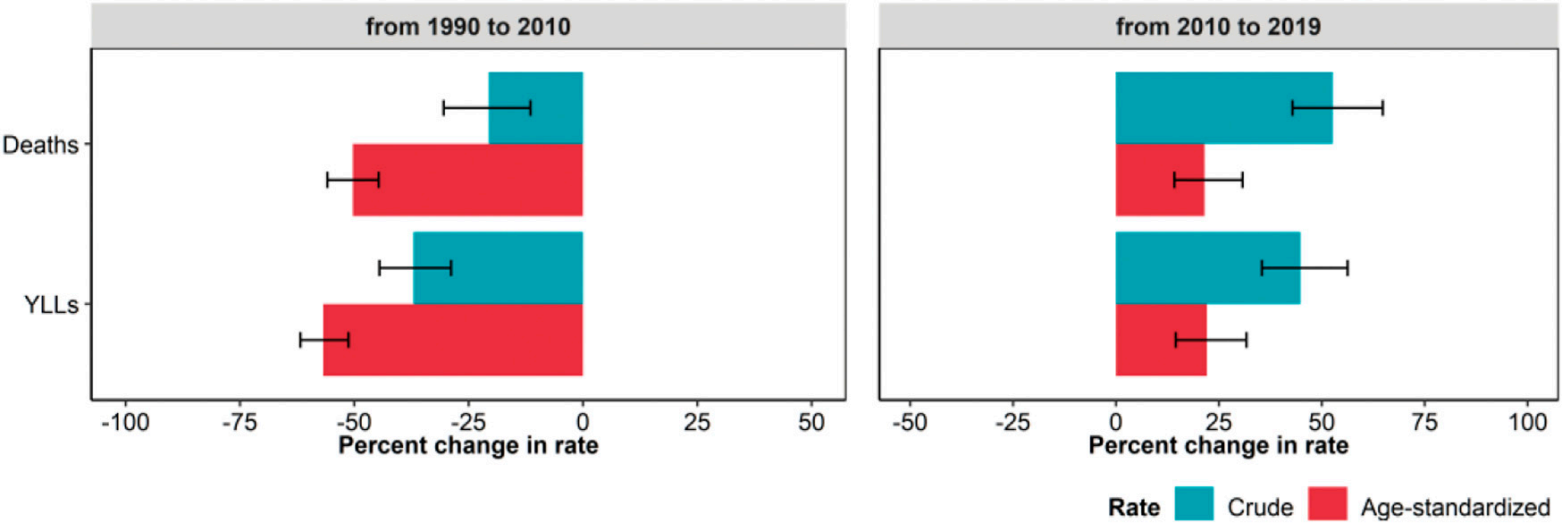
Estimated percent change in crude and age-standardized rates (per 100,000 inhabitants) of mortality (deaths), and years of life lost (YLLs*) due to ambient ozone pollution, from 1990 to 2010 and from 2010 to 2019. Whiskers represent 95% Uncertainty Intervals (Global Burden of Disease Study, Italy, 1990–2019). *The burden of ozone is limited to chronic obstructive pulmonary disease mortality, therefore YLLs account for the whole DALYs amount.

The decrease observed from 1990 to 2010 was more pronounced for age-standardized rates, while in the following period the increase was higher for crude rates (Figure 4).

Variations in the burden reflected trends in the population-weighted average ozone concentration, that oscillated from 56.6 ppb in 1990 to 48.7 ppb in 2010 to 54.1 ppb in 2019 (Supplementary Figure S1).

### Understanding the Contribution of Exposure Variation

**FIGURE 5:**
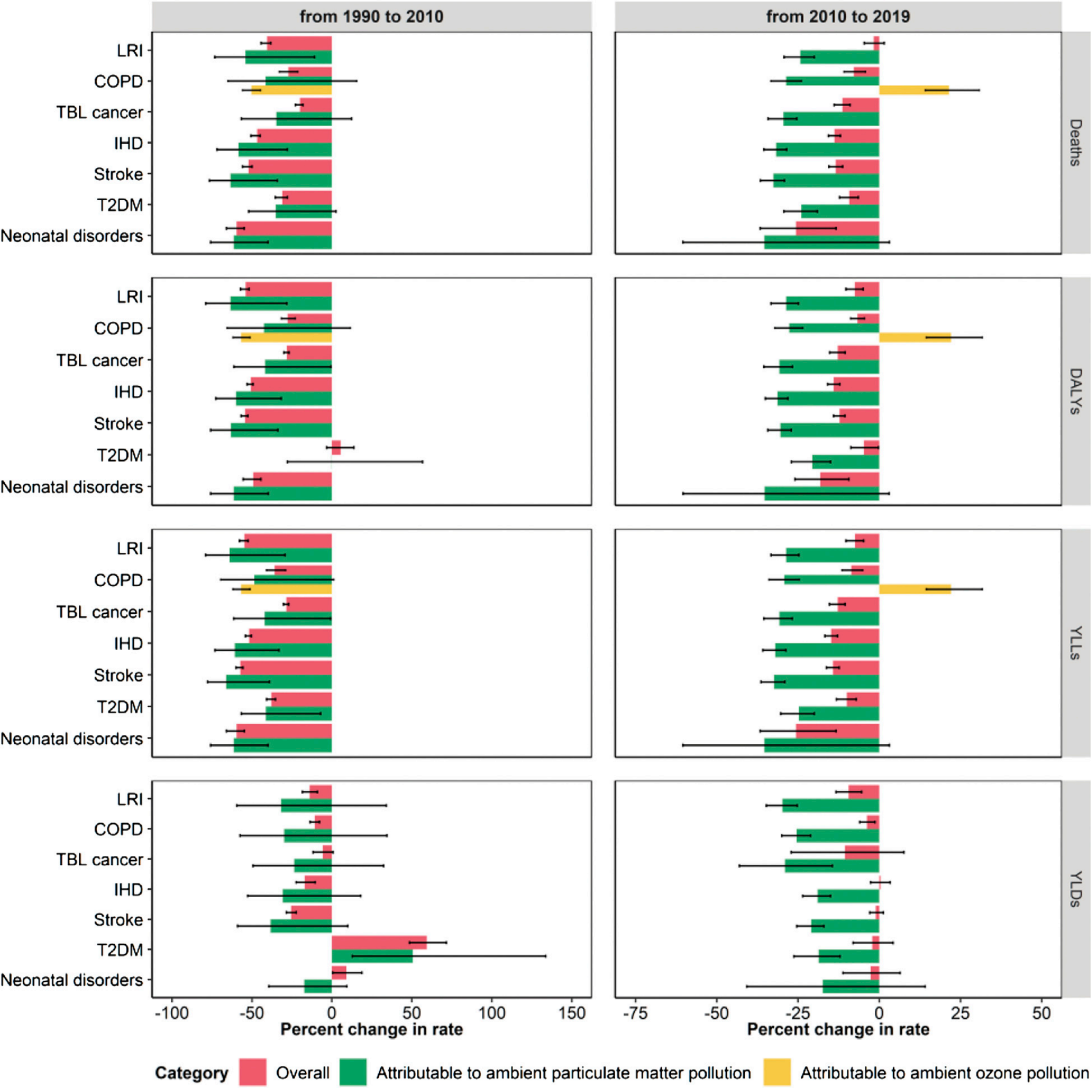
Estimated percent change in age-standardized rates (per 100,000 inhabitants) of mortality (deaths), disability adjusted life years (DALYs), years of life lost (YLLs), and years lived in disability (YLDs) for each cause associated with ambient air pollution. Comparison among overall variations and variations in rates due to ambient particulate matter and ozone pollution, from 1990 to 2010 and from 2010 to 2019. Whiskers represent 95% Uncertainty Intervals (Global Burden of Disease Study, Italy, 1990–2019). LRI, Lower respiratory infections; COPD, Chronic obstructive pulmonary disease; TBL, Tracheal, bronchus and lung; IHD, Ischemic heart disease; T2DM, Type 2 diabetes mellitus.

For all diseases, measures referred to the overall burden but YLDs displayed a significant decline during the study period, especially during 1990–2010, with the exception of T2DM (Supplementary Figures S2–S8). Indeed, during that period, reductions in age-standardized DALY rates ranged from 27.7% for COPD (95% UI: 23.0, 30.1) to 54.3% for stroke (95% UI: 52.5, 56.8). YLL and mortality rates showed similar declines, while YLD rates showed moderate declines, and two diseases, namely neonatal and T2DM, faced an increase of respectively 9.1% (95% UI: 0.5, 18.7) and 59.3% (95% UI: 48.5, 71.6) (Figure 5; Supplementary Table S4). For DALYs, YLLs and mortality, percent reductions were similar for the overall burden and for the burden attributable to ambient PM_2.5_, while the percent reduction in ozone attributable burden was significantly higher than the overall burden of COPD (Figure 5).

The temporal dynamic changed during the following period (2010–2019): percent reductions in the overall burden measures were much lower than in the previous period for all the analyzed diseases. In some cases, age-standardized DALY rate reductions were close to 0, as for T2DM (4.7%, 95% UI: 0.2, 8.7), COPD (6.7%, 95% UI: 4.6, 8.8) and LRI (7.5%, 95% UI: 4.9, 10.3) (Figure 5; Supplementary Table S4). Similar variations were traced for YLLs and mortality rates, while for most of the diseases YLDs did not decrease significantly. Conversely, the disease burden attributable to ambient PM_2.5_ consistently declined and the burden attributable to ambient ozone consistently increased (Figure 5).

## Discussion

### PM_2.5_ and Ozone Attributable Burden in 2019

GBD estimates for Italy in 2019 attribute 466,530 (95% UI: 370,621, 569,934) DALYs to ambient PM_2.5_, which ranked eighth among risk factors of the fourth hierarchical nested level of GBD Study and yielded 24,666 (95% UI: 19,177, 30,047) deaths [24]. Ozone accounted for 39,582 (95% UI: 18,282, 61,469) DALYs, with 3,487 (95% UI: 1,597, 5,385) associated deaths [24].

For both pollutants, and all burden measures (mortality, DALYs, YLLs and YLDs), attributable crude and age-adjusted rates were higher among males than in females, and age-specific rates were higher among children under 14 and in the elderly. The burden of ambient PM_2.5_, in terms of age-standardized DALY rate was unevenly stratified among exposure-related diseases: IHD and T2DM absorbed 50% of the overall burden. The gap detected between males and females is essentially explained by differences in mortality rate for these diseases [23].

The temporal variation in the burden of respiratory diseases (LRI and COPD), TBL cancer, and T2DM is due to the age-associated trends in incidence and mortality, which increase with age [25–27]. For IHD and stroke, the age-related increase comes both from age-related variations in incidence and mortality, and from the use of different exposure-response functions for each 5-year age class. This accounts for higher susceptibility to effects of air pollutants among the elderly [28, 29].

As for ozone, that is so far associated with long-term COPD mortality only, considerations mirror those related to PM_2.5_ and COPD.

### Temporal Variation in the Burden of Ambient PM_2.5_

In the period 1990–2019, we identified a clear decreasing trend in PM_2.5_-attributable burden with an overall reduction of 47.9% (95% UI: 10.3, 65.4) of the crude DALY rate. Decreases were confirmed for mortality and YLLs, but not for YLDs. Reductions in age-standardized rates were significantly higher, accounting for 65.1% (95% UI: 41.9, 76.4) in DALY rates. In the period-stratified analysis—i.e., 1990–2010 vs. 2010–2019—a significant difference between crude and age-standardized rates reductions persisted only during the second period, probably owed to the higher precision in estimates, which have narrower uncertainty intervals. These variations parallel reductions in PM_2.5_ concentrations observed after the introduction of key policy interventions, starting from 1988 [30–34]. The 2008 EU directive set a target value for PM_2.5_ of 25 μg/m^3^ for long-term concentrations, which was subsequently lowered to 20 μg/m^3^ from 1st January 2020 [31–33]. Thereafter, PM_2.5_ concentrations continued to decrease with a percentage reduction of about 37% between 1990 and 2018 [35].

The observed reduction in PM_2.5_ concentrations leads the decreasing trend detected in the related disease burden. However, reduction of exposure is only one of the drivers of the observed trend: temporal changes are also affected by demographic and epidemiologic dynamics of the population [8, 10].

The role of demographic dynamic was analyzed comparing variations of crude and age-standardized rates. The decrease observed for age-standardized rates was significantly higher than that for crude ones, meaning that the increasing proportion of elderly population counteracts the effects of exposure reduction. Worth noting is that the large difference between crude and age-standardized rates is partially explained by the different age structure of the Italian population as compared to the global one used for the standardization: in line with our observations regarding population ageing, the Italian population has a larger elderly fraction throughout the study period (Supplementary Figure S9).

We finally considered the role of the epidemiologic dynamic, which is synthesized by temporal variations in the overall burden of diseases associated with PM_2.5_. From 1990 to 2010, the overall mortality, DALY and YLL rates of the diseases of interest decreased significantly, except for T2DM. YLD rates showed a moderate decline, except for neonatal disorders and T2DM. These trends are mainly driven by improvement in case management of those conditions from the 1990s [36]. For instance, the advances in care of cardiovascular diseases and the reductions in their major risk factors, some progresses in diagnosis and management of TBL cancers, as well as novel therapeutic approaches for lower respiratory diseases effectively impacted on patient outcomes and quality of life [37–41]. As regards T2DM, there was an increase of medical check-ups and laboratory tests for early detection of T2DM and systematic glycemic control in the population at risk. Likewise, the use of novel treatments impacted on the prognosis and survival of diabetic patients [42, 43].

Proportional reductions in ambient PM_2.5_ burden closely resembled those of each investigated disease, highlighting that the reduction of PM_2.5_ burden was likely unrelated to a significant decrease in the exposure to the risk factor itself. This reflects the absence of an air quality standard for PM_2.5_, that was enforced only in late 2010 [33]. Thereafter, the overall burden of T2DM, COPD and LRI displayed only moderate reductions, and YLDs remained constant for all diseases except for LRI and COPD. PM_2.5_ burden proportional decrease was significantly higher than the overall one, suggesting that the reduction in exposure concentration had a significant role in decreasing the burden attributable to the pollutant.

If we interpret our results in the framework of an accountability study that aims at evaluating the impact of air quality regulations on public health [44], our results suggest that the national regulations introduced in Italy in 2010, establishing that the average annual concentration of PM_2.5_ should be ≤25 μg/m^3^ by 2015 [33], lead to a decrease in the average PM_2.5_ concentration (Supplementary Figure S1), that was in turn associated with a decrease in the attributable burden of air pollution-associated diseases between 2010 and 2019. These considerations encourage the enforcement of new and more stringent regulations, in line with the recently issued WHO air quality guidelines, which recommend a yearly target value of 5 μg/m^3^ for PM_2.5_ [3]. However, a proper assessment of the future impact of such interventions should carefully account for future population dynamics and their impact on air pollution-associated outcomes [45].

### Temporal Variation in the Burden of Ambient Ozone

Temporal dynamics in ozone burden differed across periods: crude and age-standardized DALY rates declined by 37.0% (95% UI: 28.9, 44.5) from 1990 to 2010, but increased by 44.8% (95% UI: 35.5, 56.3) during the following period.

This mirrors the trends in ozone concentrations, that have been increasing since 2009, despite EU Directives 2002/3/EC and 2008/50/EC set a threshold of 120 µg O_3_/m^3^ as daily maximum 8-h average not to be exceeded for more than 25 times in a year [46, 47].

When comparing the variations of crude and age-standardized DALYs and mortality rates attributable to ozone, crude rates displayed a less pronounced variation. From 1990 to 2010, the decrease in age-standardized burden was close to 50% for both DALYs and mortality, but the decrease in the crude one did not exceed 30%. The increase in age-standardized burden from 2010 to 2019 was close to 25%, but the increase in the crude one exceeded 50%. This confirms the impact of population ageing.

When comparing the variations in the overall age-standardized mortality, DALYs and YLLs of COPD, with those in ozone-attributable burden, we observed a clear indication of a beneficial impact of exposure reduction during the period 1990–2010 and an opposite detrimental effect of exposure increase in the following period. As mentioned, COPD burden was characterized by a significant reduction in both time-windows of interest. During 1990–2010, such reduction was less pronounced than the reduction in ozone-attributable burden. In 2010–2019, we observed that ozone-attributable burden consistently increased, suggesting that ozone concentrations were rising counteracting the mortality decrease.

### Strengths and Limitations

GBD estimates provide a unique opportunity to assess how air pollution has changed its impact on the health of the Italian population. Nonetheless, some limitations to our study should be mentioned. First, risk-outcome pairs identified for PM_2.5_ and ozone exclude some diseases for which evidence, although mounting, is still not strong enough, including cardiological conditions (e.g., hypertension) and neurological diseases (e.g., dementia, intellectual disability, hypertension, asthma) [8]. Worth mentioning that a preliminary global analysis on the linkage between exposures to PM_2.5_ and dementia estimated that the highest dementia-related burden attributable to air pollution was found in developed countries with aging population and moderate-to-high levels of PM_2.5_ [48]. Ostro et al. pointed out how GBD estimates are affected by variations in exposure assessment strategies and counterfactual scenarios [49], while Burnett and Cohen described how the choice of the of relative risk functions strongly impacts on the population attributable risk estimation [50]. The magnitude of risk is assumed to depend on PM_2.5_ mass alone, without considering its composition [8, 51]. Furthermore, there is no distinction between sexes, and for respiratory outcomes there is no distinction among age-classes, although susceptibility to air pollution might vary by sex and age [14]. In addition, the choice of TMREL is critical, as it significantly affects the estimated burden [51]: recent results confirm that adverse health effects of PM_2.5_ and ozone persist at levels below the current EU PM_2.5_ limits [52].

Our considerations regarding the significant role of exposure reduction are based on comparisons between estimated variations and their 95% UI, therefore they retain a high level of uncertainty, that would be reduced by carrying out a decomposition analysis, actually quantifying the estimated contributions of trends in mortality and in pollutant concentration [9]. This might be a future development of our analysis. Also, our conclusions regarding the impact of future regulatory efforts should be supported by an accountability study, that allows to infer the causal relationship between interventions and the burden of air pollution [53].

Moreover, estimates of pollutants concentrations were less reliable up to 2005, due to the lack of a monitoring system for PM_2.5_: in Italy the first monitoring stations were introduced in 2005–2006. Finally, methods used to estimate the burden did not consider the huge variations in regional concentrations of air pollutants nor the different demographic and epidemiologic dynamics of the Italian territory.

### Conclusion

In Italy, the overall decrease in the estimated burden of ambient PM_2.5_ and ozone between 1990 and 2019 suggests a beneficial effect of air quality regulations. However, while after 2010 these regulations continued to be followed by reductions in PM_2.5_ concentrations, and consequently on its attributable burden, ozone concentration is on the rise. Also, population ageing leads to an increase in susceptible population, which partially counterbalances the beneficial effects of exposure reduction. Air pollution remains a major public health concern, and new regulations are essential to mitigate its future impact. Regulatory efforts should be directed towards improving the quality of the air and protecting health, as well as to tackling climate change, which is closely interlinked with air pollution and, in particular, with ground-level ozone. Likewise, further research focusing on subnational areas and country comparisons is crucial to inform future policies and strategies to mitigate the burden.

## Data Availability

Availability of input data varies by source. Select data are available in a public, open-access repository. Select data are available on reasonable request. Select data may be obtained from a third party and are not publicly available. All results from the study are included in the article or uploaded as Supplementary Material or are available online.

## References

[1] Thurston GD Kipen H Annesi-Maesano I Balmes J Brook RD Cromar K A Joint ERS/ATS Policy Statement: what Constitutes an Adverse Health Effect of Air Pollution? an Analytical Framework. Eur Respir J (2017) 49(1):1600419. 10.1183/13993003.00419-2016 28077473 PMC5751718

[2] World Health Organization. Expert Committee on Environmental Sanitation & World Health Organization. In: Air Pollution: Fifth Report of the Expert Committee on Environmental Sanitation. Geneva, Switzerland: World Health Organization (1958). 13625723

[3] World Health Organization. WHO Global Air Quality Guidelines: Particulate Matter (PM2.5 and PM10), Ozone, Nitrogen Dioxide, Sulfur Dioxide and Carbon Monoxide. Geneva, Switzerland: World Health Organization (2021). 34662007

[4] World Health Organization – Regional Office for Europe. Review of Evidence on Health Aspects of Air Pollution – REVIHAAP Project: Technical Report. Copenhagen, Denmark: WHO Europa (2013). 27195369

[5] European Environment Agency. Directive 2008/50/EC of the European Parliament and of the Council of 21 May 2008 on Ambient Air Quality and Cleaner Air for Europe. Copenaghen: European Union (2008).

[6] United States Environmental Protection Agency. National Ambient Air Quality Standards (2020). Available from: https://www.epa.gov/criteria-air-pollutants/naaqs-table (Accessed February 22, 2023).

[7] GBD 2019 Risk Factors Collaborators. Global burden of 87 Risk Factors in 204 Countries and Territories, 1990-2019: a Systematic Analysis for the Global Burden of Disease Study 2019. Lancet (2020) 396(10258):1223–49. 10.1016/S0140-6736(20)30752-2 33069327 PMC7566194

[8] European Environment Agency. Air Pollution Due to Ozone: Health Impacts and Effects of Climate Change. European Environment Agency. (2023). Available from: https://www.eea.europa.eu/data-and-maps/indicators/air-pollution-by-ozone-2/assessment (last accessed on Feb 22, 2023).

[9] Cohen AJ Brauer M Burnett R Anderson HR Frostad J Estep K Estimates and 25-year Trends of the Global burden of Disease Attributable to Ambient Air Pollution: an Analysis of Data from the Global Burden of Diseases Study 2015. Lancet (2017) 389(10082):1907–18. 10.1016/S0140-6736(17)30505-6 28408086 PMC5439030

[10] Yin P Brauer M Cohen AJ Wang H Li J Burnett RT The Effect of Air Pollution on Deaths, Disease burden, and Life Expectancy across China and its Provinces, 1990-2017: an Analysis for the Global Burden of Disease Study 2017. Lancet Planet Health (2020) 4(9):e386–e98. 10.1016/S2542-5196(20)30161-3 32818429 PMC7487771

[11] Istituto Nazionale di Statistica (ISTAT). Invecchiamento attivo e condizioni di vita degli anziani in Italia. Roma: ISTAT (2020).

[12] Istituto Nazionale di Statistica (ISTAT). Rapporto annuale 2019: la situazione del Paese. Roma: ISTAT (2019).

[13] EUROSTAT. Ageing Europe: Looking at the Lives of Older People Inthe EU – 2019 Edition. Luxembourg: Publications Office of the European Union (2019).

[14] Yazdi MD Wang Y Di Q Requia WJ Wei Y Shi L Long-term Effect of Exposure to Lower Concentrations of Air Pollution on Mortality Among US Medicare Participants and Vulnerable Subgroups: a Doubly-Robust Approach. Lancet Planet Health (2021) 5(10):e689–e697. 10.1016/S2542-5196(21)00204-7 34627473 PMC8525655

[15] Shaffer RM Sellers SP Baker MG de Buen Kalman R Frostad J Suter MK Improving and Expanding Estimates of the Global burden of Disease Due to Environmental Health Risk Factors. Environ Health Perspect (2019) 127(10):105001. 10.1289/EHP5496 31626566 PMC6867191

[16] Stevens GA Alkema L Black RE Boerma JT Collins GS Ezzati M Guidelines for Accurate and Transparent Health Estimates Reporting: the GATHER Statement. Lancet (2016) 388(10062):e19–e23. 10.1016/S0140-6736(16)30388-9 27371184

[17] van Donkelaar A Martin RV Brauer M Hsu NC Kahn RA Levy RC Global Estimates of Fine Particulate Matter Using a Combined Geophysical-Statistical Method with Information from Satellites, Models, and Monitors. Environ Sci Technol (2016) 50(7):3762–72. 10.1021/acs.est.5b05833 26953851

[18] Istituto Nazionale di Statistica (ISTAT). Health for All - Italia 2020 (2023). Available from: https://www.istat.it/it/archivio/14562 (last accessed on Feb 22, 2023).

[19] Zheng P Barber R Sorensen RJD Murray CJL Aravkin AY. Trimmed Constrained Mixed Effects Models: Formulations and Algorithms. J Comput Graphical Stat (2021) 30(3):544–56. 10.1080/10618600.2020.1868303

[20] Global Burden of Disease Collaborative Network. Global Burden of Disease Study 2019 (GBD 2019) Particulate Matter Risk Curves. Seattle, United States of America: Institute for Health Metrics and Evaluation (2021). 10.6069/KHWH-2703

[21] Global Burden of Disease Project. Global Health Data Exchange - GBD Results Tool (2023). Available from: http://ghdx.healthdata.org/gbd-results-tool (last accessed on Feb 22, 2023).

[22] State of Global Air. State of Global Air (2020). Available from: https://www.stateofglobalair.org/data/#/air/plot ((last accessed on Mar 8, 2022).

[23] GBD 2019 Diseases and Injuries Collaborators. Global burden of 369 Diseases and Injuries in 204 Countries and Territories, 1990-2019: a Systematic Analysis for the Global Burden of Disease Study 2019. Lancet (2020) 396(10258):1204–22. 10.1016/S0140-6736(20)30925-9 33069326 PMC7567026

[24] Global Burden of Disease Project. Global Health Data Exchange - GBD Compare (2023). Available from: https://vizhub.healthdata.org/gbd-compare/ (last accessed on Feb 22, 2023).

[25] GBD 2016 Lower Respiratory Infections Collaborators. Estimates of the Global, Regional, and National Morbidity, Mortality, and Aetiologies of Lower Respiratory Infections in 195 Countries, 1990–2016: a Systematic Analysis for the Global Burden of Disease Study 2016. Lancet Infect Dis (2018) 18(11):1191–210. 10.1016/S1473-3099(18)30310-4 30243584 PMC6202443

[26] Global Burden of Disease Cancer Collaboration Fitzmaurice C Abate D Abbasi N Abbastabar H Abd-Allah F Global, Regional, and National Cancer Incidence, Mortality, Years of Life Lost, Years Lived with Disability, and Disability-Adjusted Life-Years for 29 Cancer Groups, 1990 to 2017. A Systematic Analysis for the Global Burden of Disease Study. JAMA Oncol (2019) 5(12):1749–68. 10.1001/jamaoncol.2019.2996 31560378 PMC6777271

[27] Munshi MN Meneilly GS Rodríguez-Mañas L Close KL Conlin PR Cukierman-Yaffe T Diabetes in Ageing: Pathways for Developing the Evidence Base for Clinical Guidance. Lancet Diabetes Endocrinol (2020) 8(10):855–67. 10.1016/S2213-8587(20)30230-8 32946822 PMC8223534

[28] Boezen HM Vonk JM van der Zee SC Gerritsen J Hoek G Brunekreef B Susceptibility to Air Pollution in Elderly Males and Females. Eur Respir J (2005) 25:1018–24. 10.1183/09031936.05.00076104 15929956

[29] Aga E Samoli E Touloumi G Anderson HR Cadum E Forsberg B Short-term Effects of Ambient Particles on Mortality in the Elderly: Results from 28 Cities in the APHEA2 Project. Eur Respir J Suppl (2003) 40:28s–33s. 10.1183/09031936.03.00402803 12762571

[30] European Environment Agency. Urban PM2.5 Concentrations Presented as Multi-Annual Average in the EU, 2008–2010. EEA (2012). Available from: https://www.eea.europa.eu/data-and-maps/figures/urban-pm2.5-concentrations-presented-as (last accessed on Feb 22, 2022).

[31] Organisation for Economic Co-operation and Development (OECD). Air Pollution Exposure. OECD: OECD data (2020). Available from: https://data.oecd.org/air/air-pollution-exposure.htm ((last accessed on Feb 22, 2023).

[32] Istituto superiore per la protezione e la ricerca ambientale (ISPRA). Analisi dei trend dei principali inquinanti atmosferici in Italia (2008 – 2017). Roma: ISPRA (2018). Available from: https://www.isprambiente.gov.it/files2019/pubblicazioni/rapporti/R_302_18_TREND_ARIA.pdf ((last accessed on Feb 22, 2023).

[33] Gazzetta Ufficiale. Decreto Legislativo 13 agosto 2010, n.155 “Attuazione della direttiva 2008/50/CE relativa alla qualità dell'aria ambiente e per un'aria più pulita in Europa” (2010). Gazzetta Ufficiale della Repubblica Italiana n. 216 del 15. Rome: stituto Poligrafico e Zecca dello Stato.

[34] Viviano G Settimo G. Normativa sulla qualità dell'aria e recepimento delle direttive della Unione Europea. Ann Ist Super Sanità (2003) 39(3):343–50. 15098554

[35] Istituto superiore per la protezione e la ricerca ambientale (ISPRA). Italian Emission Inventory 1990-2018. Informative Inventory Report 2020. Roma: ISPRA (2020). Available from: https://www.isprambiente.gov.it/it/pubblicazioni/rapporti/inventario-nazionale-delle-emissioni-in-atmosfera-1990-2018.-informative-inventory-report-2020 ((last accessed on Feb 22, 2022).

[36] GBD 2017 Italy Collaborators. Italy's Health Performance, 1990-2017: Findings from the Global Burden of Disease Study 2017. Lancet Public Health (2019) 4(12):e645–e657. 10.1016/S2468-2667(19)30189-6 31759893 PMC7098474

[37] Cortesi PA Fornari C Madotto F Conti S Naghavi M Bikbov B Trends in Cardiovascular Diseases burden and Vascular Risk Factors in Italy: The Global Burden of Disease Study 1990-2017. Eur J Prev Cardiol (2020) 28:385–96. 10.1177/2047487320949414 33966080

[38] Palmieri L Bennett K Giampaoli S Capewell S. Explaining the Decrease in Coronary Heart Disease Mortality in Italy between 1980 and 2000. Am J Public Health (2010) 100(4):684–92. 10.2105/AJPH.2008.147173 19608958 PMC2836342

[39] Santalucia P Baviera M Cortesi L Tettamanti M Marzona I Nobili A Epidemiologic Trends in Hospitalized Ischemic Stroke from 2002 to 2010: Results from a Large Italian Population-Based Study. J Stroke Cerebrovasc Dis (2015) 24(8):1917–23. 10.1016/j.jstrokecerebrovasdis.2015.05.008 26051662

[40] Associazione Italiana di Oncologia Medica. I Numeri del Cancro in Italia 2020. Milano: AIOM (2020). Available from: https://www.aiom.it/wp-content/uploads/2020/10/2020_Numeri_Cancro-operatori_web.pdf ((last accessed on Feb 22, 2023).

[41] Pesce G. Mortality Rates for Chronic Lower Respiratory Diseases in Italy from 1979 to 2010: an Age–Period–Cohort Analysis. ERJ Open Res (2016) 2:00093–2015. 10.1183/23120541.00093-2015 PMC5005165 27730182

[42] Istituto nazionale di statistica (ISTAT). Il Diabete in Italia – Anni 2000-2011. Rome: ISTAT (2012). Available from: https://www.istat.it/it/files//2012/09/Il-diabete-in-Italia.pdf ((last accessed on Feb 22, 2023).

[43] Istituto nazionale di statistica (ISTAT). Il Diabete in Italia – Anni 2000-2016. Rome: ISTAT (2017). Available from: https://www.istat.it/it/files/2017/07/REPORT_DIABETE.pdf ((last accessed on Feb 22, 2023).

[44] Boogaard H van Erp AM Walker KD Shaikh R. Accountability Studies on Air Pollution and Health: the HEI Experience. Curr Environ Health Rep (2017) 4(4):514–22. 10.1007/s40572-017-0161-0 28988407

[45] GBD MAPS Working Group. Burden of Disease Attributable to Coal-Burning and Other Major Sources of Air Pollution in China. Special Report 20. Boston, MA: Health Effects Institute (2016).

[46] European Environment Agency. Air Quality in Europe - 2020 Report. Luxembourg: Publications Office of the European Union (2020).

[47] Gazzetta Ufficiale. Decreto Legislativo 21 Maggio 2004, n. 183 “Attuazione Della Direttiva 2002/3/CE Relativa All'ozono Nell'aria” (2004). Gazzetta Ufficiale della Repubblica Italiana n. 171 del 23. Rome: stituto Poligrafico e Zecca dello Stato.

[48] Ru M Brauer M Lamarque JF Shindell D. Exploration of the Global Burden of Dementia Attributable to PM2.5: What Do We Know Based on Current Evidence? Geohealth (2021) 5(5):e2020GH000356. 10.1029/2020GH000356 PMC8143277 34084981

[49] Ostro B Spadaro JV Gumy S Mudu P Awe Y Forastiere F Assessing the Recent Estimates of the Global burden of Disease for Ambient Air Pollution: Methodological Changes and Implications for Low- and Middle-Income Countries. Environ Res (2018) 166:713–25. 10.1016/j.envres.2018.03.001 29880237

[50] Burnett R Cohen A. Relative Risk Functions for Estimating Excess Mortality Attributable to Outdoor PM2.5 Air Pollution: Evolution and State-Of-The-Art. Atmosphere (2020) 11(6):589. 10.3390/atmos11060589

[51] Boogaard H Walker K Cohen AJ. Air Pollution: the Emergence of a Major Global Health Risk Factor. Int Health (2019) 11(6):417–21. 10.1093/inthealth/ihz078 31613318

[52] Di Q Wang Y Zanobetti A Koutrakis P Choirat C Dominici F Air Pollution and Mortality in the Medicare Population. N Engl J Med (2017) 376(26):2513–22. 10.1056/NEJMoa1702747 28657878 PMC5766848

[53] Zigler CM Kim C Choirat C Hansen JB Wang Y Hund L Causal Inference Methods for Estimating Long-Term Health Effects of Air Quality Regulations. Research Report 187. Boston, MA: Health Effects Institute (2016). 27526497

