# Corrigendum: Time-Trends in Air Pollution Impact on Health in Italy, 1990–2019: An Analysis from the Global Burden of Disease Study 2019

**DOI:** 10.3389/ijph.2024.1607320

**Published:** 2024-05-16

**Authors:** Sara Conti, Carla Fornari, Pietro Ferrara, Ippazio C. Antonazzo, Fabiana Madotto, Eugenio Traini, Miriam Levi, Achille Cernigliaro, Benedetta Armocida, Nicola L. Bragazzi, Ennio Cadum, Michele Carugno, Giacomo Crotti, Silvia Deandrea, Paolo A. Cortesi, Davide Guido, Ivo Iavicoli, Sergio Iavicoli, Carlo La Vecchia, Paolo Lauriola, Paola Michelozzi, Salvatore Scondotto, Massimo Stafoggia, Francesco S. Violante, Cristiana Abbafati, Luciana Albano, Francesco Barone-Adesi, Antonio Biondi, Cristina Bosetti, Danilo Buonsenso, Giulia Carreras, Giulio Castelpietra, Alberico Catapano, Maria S. Cattaruzza, Barbara Corso, Giovanni Damiani, Francesco Esposito, Silvano Gallus, Davide Golinelli, Simon I. Hay, Gaetano Isola, Caterina Ledda, Stefania Mondello, Paolo Pedersini, Umberto Pensato, Norberto Perico, Giuseppe Remuzzi, Francesco Sanmarchi, Rocco Santoro, Biagio Simonetti, Brigid Unim, Marco Vacante, Massimiliano Veroux, Jorge H. Villafañe, Lorenzo Monasta, Lorenzo G. Mantovani

**Affiliations:** ^1^ Research Center on Public Health, University of Milan Bicocca, Monza, Italy; ^2^ Laboratory of Public Health, Auxologico Research Hospital—IRCCS, Milan, Italy; ^3^ Dipartimento di Anestesia, Rianimazione ed Emergenza Urgenza, Fondazione IRCCS Ca’ Granda Ospedale Maggiore Policlinico, Milano, Italy; ^4^ Institute for Risk Assessment Sciences (IRAS), Utrecht University, Utrecht, Netherlands; ^5^ Epidemiology Unit, Department of Prevention, Central Tuscany Local Health Authority, Florence, Italy; ^6^ Health Activities and Epidemiological Observatory Department, Health Authority Sicily Region, Parlemo, Italy; ^7^ Clinical Pathology Complex Hospital Unit, Health Authority Trapani Province, Trapani, Italy; ^8^ Department of Cardiovascular, Endocrine-Metabolic Disease and Aging, National Institute of Health (ISS), Rome, Italy; ^9^ Laboratory for Industrial and Applied Mathematics (LIAM), Department of Mathematics and Statistics, Faculty of Science, York University, Toronto, ON, Canada; ^10^ Department of Hygiene and Health Prevention and Complex Operative Unit Environmental Health and Innovative Projects, Health Protection Agency, Pavia, Italy; ^11^ Department of Clinical Sciences and Community Health, University of Milan, Milan, Italy; ^12^ Epidemiology Unit, Fondazione IRCCS Ca’ Granda Ospedale Maggiore Policlinico, Milan, Italy; ^13^ Servizio Epidemiologico Aziendale, Agenzia di Tutela della Salute di Bergamo, Bergamo, Italy; ^14^ Neurology, Public Health and Disability Unit, Carlo Besta Neurological Institute, Fondazione IRCCS Istituto Neurologico Carlo Besta, Milan, Italy; ^15^ Department of Public Health, University of Naples Federico II, Naples, Italy; ^16^ Department of Occupational and Environmental Medicine Epidemiology and Hygiene, Italian National Workers Compensation Authority (IIL), Monteporzio Catone, Italy; ^17^ International Network of Public Health and Environment Tracking (INPHET), Milan, Italy; ^18^ Department of Epidemiology, Lazio Region Health Authority (ASL RM1), Rome, Italy; ^19^ Occupational Health Unit, Department of Medical and Surgical Sciences, University of Bologna, Bologna, Italy; ^20^ Department of Juridical and Economic Studies, Faculty of Law, Sapienza University of Rome, Rome, Italy; ^21^ Department of Experimental Medicine, University of Campania Luigi Vanvitelli, Naples, Italy; ^22^ Department of Translational Medicine, University of Eastern Piedmont, Novara, Italy; ^23^ Department of General Surgery and Medical-Surgical Specialties, University of Catania, Catania, Italy; ^24^ Department of Oncology, Mario Negri Pharmacological Research Institute (IRCCS), Milano, Italy; ^25^ Department of Woman and Child Health and Public Health, Fondazione Policlinico Universitario A. Gemelli IRCCS (Agostino Gemelli University Polyclinic IRCCS), Rome, Italy; ^26^ Global Health Research Institute, Università Cattolica del Sacro Cuore (Catholic University of Sacred Heart), Rome, Italy; ^27^ Institute for Cancer Research, Prevention and Clinical Network (ISPRO), Florence, Italy; ^28^ Department of Medicine, University of Udine, Udine, Italy; ^29^ Department of Mental Health, Healthcare Agency “Friuli Occidentale”, Pordenone, Italy; ^30^ Department of Pharmacological and Biomolecular Sciences, University of Milan, Milan, Italy; ^31^ MultiMedica, IRCCS, Sesto SanGiovanni, Italy; ^32^ Department of Public Health and Infectious Diseases, Sapienza University of Rome, Rome, Italy; ^33^ Institute of Neuroscience, National Research Council (CNR), Pisa, Italy; ^34^ IRCCS Istituto Ortopedico Galeazzi (Galeazzi Orthopedic Institute IRCCS), University of Milan, Milan, Italy; ^35^ Department of Dermatology, Case Western Reserve University, Cleveland, OH, United States; ^36^ Department of Biomedical and Neuromotor Sciences, University of Bologna, Bologna, Italy; ^37^ Department of Environmental Health Sciences, Mario Negri Institute for Pharmacological Research, Milan, Italy; ^38^ Institute for Health Metrics and Evaluation, University of Washington, Seattle, WA, United States; ^39^ Department of Health Metrics Sciences, School of Medicine, University of Washington, Seattle, WA, United States; ^40^ Department of Clinical and Experimental Medicine, University of Catania, Catania, Italy; ^41^ Department of Biomedical and Dental Sciences and Morphofunctional Imaging, Messina University, Messina, Italy; ^42^ Clinical Research Department, IRCCS Fondazione Don Carlo Gnocchi, Milan, Italy; ^43^ Department of Neurology, IRCCS Humanitas Research Hospital, Milan, Italy; ^44^ Istituto di Ricerche Farmacologiche Mario Negri IRCCS, Bergamo, Italy; ^45^ Daccude, Todi, Italy; ^46^ Department of Law, Economics, Management and Quantitative Methods, University of Sannio, Benevento, Italy; ^47^ WSB University in Gdańsk, Gdańsk, Poland; ^48^ Department of Medical, Surgical Sciences and Advanced Technologies, University of Catania, Catania, Italy; ^49^ Clinical Epidemiology and Public Health Research Unit, Burlo Garofolo Institute for Maternal and Child Health, Trieste, Italy

**Keywords:** air pollution, particulate matter, ozone, global burden of disease, air quality regulations

There was an error regarding the affiliation for Paolo Lauriola. The affiliation 17 “International Society Doctors for the Environment, Milan, Italy,” should be replaced with “International Network of Public Health and Environment Tracking (INPHET).”

The authors apologize for this error and state that this does not change the scientific conclusions of the article in any way. The first published incorrect version of the article has been updated.

