# Supplementary material for: Time-Trends in Air Pollution Impact on Health in Italy, 1990–2019: An Analysis From the Global Burden of Disease Study 2019
Source: Int J Public Health. 2023 Jun 2;68:1605959. doi: 10.3389/ijph.2023.1605959 (PMC10280378; doi:10.3389/ijph.2023.1605959)

**Time-Trends in Air Pollution Impact on Health in Italy, 1990–2019: An Analysis From the Global Burden of Disease Study 2019**

**Supplementary material**

**Table S1.** Crude and age-standardized mortality rates due to ambient particulate matter and ozone pollution (Italy, 2019) and temporal change (Italy, 1990–2019)

|  | **Crude mortality rate per 100,000 inhabitants** | | | | **Age-standardized mortality rate per 100,000 inhabitants** | | | |
| --- | --- | --- | --- | --- | --- | --- | --- | --- |
|  | **Estimate (95% UI)**  **for 2019** | **Percent change (95% UI)** | | | **Estimate (95% UI)**  **for 2019** | **Percent change (95% UI)** | | |
|  |  | **from 1990 to 2010** | **from 2010 to 2019** | **from 1990 to 2019** |  | **from 1990 to 2010** | **from 2010 to 2019** | **from 1990 to 2019** |
| **Ambient particulate matter pollution** |  |  |  |  |  |  |  |  |
| **Total** | 40.90 (31.80,49.82) | -31.3 (-54.3,21.0) | -15.4 (-19.9,-11.5) | -41.9 (-62.1,3.2) | 14.79 (11.79,17.89) | -53.5 (-68.7,-19.3) | -30.2 (-33.7,-27.3) | -67.5 (-78.8,-43.1) |
| **Sex** |  |  |  |  |  |  |  |  |
| Males | 45.21 (36.14,54.80) | -33.4 (-55.3,16.0) | -17.0 (-21.5,-13.1) | -44.8 (-63.8,-3.2) | 20.24 (16.27,24.44) | -52.9 (-68.5,-18.3) | -32.4 (-36.0,-29.4) | -68.1 (-79.3,-44.4) |
| Females | 36.81 (27.87,45.38) | -28.6 (-52.7,26.3) | -13.4 (-18.4,-9.1) | -38.2 (-59.9,9.7) | 10.54 (8.18,12.84) | -54.2 (-69.3,-20.2) | -28.5 (-32.6,-25.2) | -67.3 (-78.5,-42.5) |
| **Age class** |  |  |  |  |  |  |  |  |
| Under 5 | 1.66 (1.11,2.31) | -65.3 (-77.4,-47.9) | -38.7 (-61.4,-4.5) | -78.7 (-87.0,-67.1) |  |  |  |  |
| 5-14 | 0.01 (0.01,0.02) | -72.8 (-84.1,-45.5) | -38.1 (-44.4,-30.8) | -83.1 (-90.3,-65.8) |  |  |  |  |
| 15-49 | 1.95 (1.56,2.41) | -46.2 (-62.6,-10.0) | -29.0 (-33.2,-25.1) | -61.8 (-73.9,-35.9) |  |  |  |  |
| 50-74 | 16.70 (13.32,20.43) | -55.4 (-70.1,-23.3) | -32.8 (-36.7,-29.8) | -70.0 (-80.3,-48.3) |  |  |  |  |
| 75 plus | 158.14 (119.93,196.88) | -46.5 (-65.1,-3.8) | -26.1 (-30.0,-22.8) | -60.5 (-74.5,-28.5) |  |  |  |  |
| **Cause** |  |  |  |  |  |  |  |  |
| Ischemic heart disease | 13.47 (10.19,17.09) | -39.3 (-58.9,5.3) | -17.3 (-22.4,-12.6) | -49.9 (-67.1,-12.0) | 4.92 (3.81,6.12) | -58.4 (-71.8,-27.9) | -31.8 (-35.5,-28.5) | -71.6 (-81.1,-50.7) |
| Stroke | 9.23 (7.07,11.58) | -44.7 (-64.6,0.8) | -17.6 (-22.9,-12.8) | -54.4 (-71.7,-16.9) | 3.16 (2.47,3.92) | -63.5 (-76.6,-34.1) | -32.6 (-36.6,-29.2) | -75.4 (-84.6,-55.7) |
| Tracheal, bronchus, and lung cancer | 6.94 (4.92,9.42) | -12.5 (-41.7,51.0) | -19.3 (-24.8,-14.7) | -29.5 (-53.6,23.5) | 2.88 (2.04,3.91) | -34.8 (-56.6,12.4) | -29.5 (-34.2,-25.4) | -54.0 (-69.8,-19.3) |
| Chronic obstructive pulmonary disease | 4.77 (3.16,6.56) | -6.7 (-43.8,84.8) | -10.2 (-16.2,-4.1) | -16.2 (-50.9,65.4) | 1.51 (1.00,2.07) | -41.4 (-64.9,15.6) | -28.7 (-33.3,-24.0) | -58.2 (-75.4,-17.5) |
| Type 2 diabetes mellitus | 4.74 (3.00,6.61) | -3.3 (-29.0,52.9) | -7.2 (-13.9,-1.2) | -10.2 (-36.9,43.6) | 1.61 (1.03,2.24) | -35.2 (-51.9,2.5) | -24.0 (-29.3,-19.0) | -50.7 (-64.9,-22.1) |
| Lower respiratory infections | 1.67 (0.97,2.59) | -19.8 (-53.0,57.8) | -0.4 (-7.0,5.2) | -20.1 (-54.5,58.0) | 0.53 (0.31,0.83) | -54.1 (-73.3,-10.8) | -24.3 (-29.3,-20.0) | -65.2 (-79.9,-32.5) |
| Neonatal disorders | 0.06 (0.04,0.09) | -63.5 (-77.1,-43.1) | -48.7 (-68.5,-18.2) | -81.3 (-88.6,-70.0) | 0.17 (0.11,0.24) | -61.4 (-75.8,-39.9) | -35.4 (-60.4,3.1) | -75.1 (-84.9,-60.0) |
|  |  |  |  |  |  |  |  |  |
| **Ambient ozone pollution** |  |  |  |  |  |  |  |  |
| **Total** | 5.78 (2.65,8.93) | -20.6 (-30.4,-11.5) | 52.6 (42.9,64.8) | 21.2 (6.7,33.1) | 1.83 (0.84,2.83) | -50.3 (-55.9,-44.7) | 21.5 (14.2,30.8) | -39.6 (-45.2,-34.2) |
| **Sex** |  |  |  |  |  |  |  |  |
| Males | 6.98 (3.22,10.76) | -31.0 (-38.6,-23.2) | 50.9 (39.6,64.1) | 4.1 (-6.0,14.5) | 2.91 (1.34,4.48) | -54.6 (-59.4,-49.7) | 15.1 (6.7,25.0) | -47.7 (-52.3,-42.8) |
| Females | 4.64 (2.07,7.25) | 1.8 (-14.8,15.8) | 55.0 (42.6,69.5) | 57.8 (29.1,77.7) | 1.17 (0.53,1.86) | -39.8 (-47.0,-32.0) | 24.6 (16.7,35.5) | -25.0 (-33.7,-16.2) |
| **Age class** |  |  |  |  |  |  |  |  |
| 15-49 | 0.05 (0.02,0.08) | -44.5 (-53.2,-33.1) | 33.1 (20.9,49.1) | -26.1 (-35.9,-10.2) |  |  |  |  |
| 50-74 | 1.24 (0.57,1.92) | -61.4 (-66.4,-54.1) | 20.1 (10.8,31.6) | -53.6 (-58.7,-45.7) |  |  |  |  |
| 75 plus | 26.52 (12.06,40.95) | -40.5 (-48.0,-34.0) | 29.4 (20.9,40.0) | -23.0 (-31.9,-15.6) |  |  |  |  |

* The burden of ozone is limited to mortality for chronic obstructive pulmonary disease among people aged 15 and more. UI: Uncertainty Interval

**Table S2.** Crude and age-standardized rates of years of life lost (YLLs) due to ambient particulate matter and ozone pollution (Italy, 2019) and temporal change (Italy, 1990–2019)

|  | **Crude YLLs rate per 100,000 inhabitants** | | | | **Age-standardized YLLs rate per 100,000 inhabitants** | | | |
| --- | --- | --- | --- | --- | --- | --- | --- | --- |
|  | **Estimate (95% UI)**  **for 2019** | **Percent change (95% UI)** | | | **Estimate (95% UI)**  **for 2019** | **Percent change (95% UI)** | | |
|  |  | **from 1990 to 2010** | **from 2010 to 2019** | **from 1990 to 2019** |  | **from 1990 to 2010** | **from 2010 to 2019** | **from 1990 to 2019** |
| **Ambient particulate matter pollution** |  |  |  |  |  |  |  |  |
| **Total** | 590.51 (475.59,712.63) | -43.5 (-61.6,-4.1) | -21.1 (-25.2,-17.6) | -55.4 (-70.5,-22.6) | 269.75 (220.04,320.51) | -57.2 (-70.7,-29.6) | -31.1 (-35.1,-27.8) | -70.5 (-80.1,-51.1) |
| **Sex** |  |  |  |  |  |  |  |  |
| Males | 736.07 (596.53,890.17) | -44.8 (-62.5,-7.1) | -22.7 (-27.0,-19.1) | -57.3 (-71.6,-27.2) | 376.30 (307.01,449.35) | -57.6 (-71.1,-30.2) | -33.1 (-37.2,-29.6) | -71.6 (-80.8,-52.8) |
| Females | 452.62 (352.38,551.50) | -41.2 (-60.3,0.7) | -18.5 (-23.3,-14.6) | -52.1 (-68.4,-16.6) | 177.77 (143.61,212.34) | -56.9 (-70.2,-28.0) | -28.7 (-34.3,-23.5) | -69.3 (-79.0,-50.4) |
| **Age class** |  |  |  |  |  |  |  |  |
| Under 5 | 147.57 (98.52,205.03) | -65.2 (-77.4,-47.8) | -38.7 (-61.4,-4.5) | -78.7 (-87.0,-67.0) |  |  |  |  |
| 5-14 | 0.78 (0.46,1.20) | -72.7 (-84.2,-45.5) | -38.3 (-44.6,-31.0) | -83.2 (-90.4,-65.9) |  |  |  |  |
| 15-49 | 89.36 (71.49,110.55) | -47.1 (-63.3,-11.6) | -29.5 (-33.6,-25.7) | -62.7 (-74.7,-37.5) |  |  |  |  |
| 50-74 | 411.97 (329.14,503.47) | -56.7 (-70.8,-25.9) | -32.6 (-36.4,-29.6) | -70.8 (-80.8,-49.8) |  |  |  |  |
| 75 plus | 1,571.42 (1,204.42,1,940.50) | -52.4 (-68.6,-14.8) | -29.7 (-33.5,-26.5) | -66.5 (-78.4,-39.7) |  |  |  |  |
| **Cause** |  |  |  |  |  |  |  |  |
| Ischemic heart disease | 200.02 (156.19,248.14) | -49.5 (-65.5,-13.7) | -22.6 (-27.0,-18.6) | -60.9 (-73.8,-31.9) | 90.49 (71.68,111.13) | -60.8 (-73.1,-33.1) | -32.0 (-35.8,-28.7) | -73.3 (-82.0,-54.4) |
| Tracheal, bronchus, and lung cancer | 128.51 (91.21,173.91) | -27.6 (-51.8,24.4) | -22.5 (-27.8,-17.9) | -43.9 (-63.1,-1.6) | 60.93 (43.13,82.28) | -42.1 (-61.3,-0.8) | -30.8 (-35.5,-26.8) | -59.9 (-73.7,-30.1) |
| Stroke | 122.28 (97.06,150.81) | -54.2 (-70.0,-17.5) | -22.6 (-27.2,-18.5) | -64.5 (-77.5,-36.9) | 51.40 (41.19,62.61) | -66.1 (-77.8,-39.0) | -32.4 (-36.4,-29.0) | -77.1 (-85.3,-59.1) |
| Type 2 diabetes mellitus | 61.83 (39.40,86.23) | -20.4 (-41.1,26.6) | -12.6 (-18.9,-6.9) | -30.4 (-50.7,10.6) | 24.37 (15.59,34.00) | -41.4 (-56.6,-6.9) | -24.7 (-30.3,-20.0) | -55.8 (-68.6,-30.2) |
| Chronic obstructive pulmonary disease | 54.10 (35.90,74.53) | -25.4 (-55.4,48.4) | -15.6 (-21.3,-10.2) | -37.1 (-62.9,24.0) | 19.39 (12.80,27.14) | -48.6 (-69.5,1.2) | -29.2 (-33.9,-24.6) | -63.6 (-78.6,-29.1) |
| Lower respiratory infections | 18.27 (10.83,28.19) | -40.7 (-65.2,15.9) | -10.5 (-16.5,-5.3) | -46.9 (-69.4,4.9) | 7.69 (4.62,11.79) | -64.0 (-79.0,-29.2) | -28.6 (-33.3,-24.7) | -74.3 (-84.8,-50.1) |
| Neonatal disorders | 5.44 (3.56,7.64) | -63.5 (-77.1,-43.1) | -48.7 (-68.5,-18.2) | -81.3 (-88.6,-70.0) | 15.33 (10.00,21.51) | -61.4 (-75.8,-39.9) | -35.4 (-60.4,3.1) | -75.1 (-84.9,-60.0) |
|  |  |  |  |  |  |  |  |  |
| **Ambient ozone pollution** |  |  |  |  |  |  |  |  |
| **Total** | 65.63 (30.31,101.92) | -37.0 (-44.5,-28.9) | 44.8 (35.5,56.3) | -8.8 (-18.6,0.4) | 23.56 (10.98,36.79) | -56.8 (-61.8,-51.3) | 22.1 (14.5,31.7) | -47.3 (-52.3,-41.1) |
| **Sex** |  |  |  |  |  |  |  |  |
| Males | 83.77 (38.92,129.07) | -44.6 (-50.9,-37.8) | 42.9 (32.3,55.7) | -20.9 (-28.6,-12.5) | 36.02 (16.73,55.31) | -61.1 (-65.5,-56.3) | 15.8 (7.7,26.3) | -55.0 (-59.1,-50.4) |
| Females | 48.44 (22.56,76.41) | -18.0 (-29.4,-6.0) | 47.8 (35.8,61.7) | 21.2 (2.9,37.5) | 14.82 (6.82,23.66) | -45.3 (-52.2,-36.1) | 26.4 (17.8,37.9) | -30.9 (-38.4,-20.6) |
| **Age class** |  |  |  |  |  |  |  |  |
| 15-49 | 2.30 (1.06,3.57) | -45.4 (-53.8,-34.0) | 32.0 (19.9,47.9) | -27.9 (-37.4,-12.2) |  |  |  |  |
| 50-74 | 28.30 (13.22,44.01) | -62.6 (-67.5,-55.7) | 20.7 (11.3,32.3) | -54.9 (-59.9,-47.1) |  |  |  |  |
| 75 plus | 251.35 (114.29,389.58) | -48.4 (-54.7,-42.7) | 23.4 (15.1,33.8) | -36.4 (-43.6,-30.1) |  |  |  |  |

* The burden of ozone is limited to mortality for chronic obstructive pulmonary disease among people aged 15 and more. UI: Uncertainty Interval

**Table S3.** Crude and age-standardized rates of years lived in disability (YLDs) due to ambient particulate matter pollution (Italy, 2019) and temporal change (Italy, 1990–2019)

|  | **Crude YLDs rate per 100,000 inhabitants** | | | | **Age-standardized YLDs rate per 100,000 inhabitants** | | | |
| --- | --- | --- | --- | --- | --- | --- | --- | --- |
|  | **Estimate (95% UI)**  **for 2019** | **Percent change (95% UI)** | | | **Estimate (95% UI)**  **for 2019** | **Percent change (95% UI)** | | |
|  |  | **from 1990 to 2010** | **from 2010 to 2019** | **from 1990 to 2019** |  | **from 1990 to 2010** | **from 2010 to 2019** | **from 1990 to 2019** |
| **Ambient particulate matter pollution** |  |  |  |  |  |  |  |  |
| **Total** | 183.00 (117.16 , 258.73) | 31.0 (-10.7 , 121.0) | -13.5 (-19.3 , -8.5) | 13.3 (-25.2 , 92.9) | 87.74 (56.10 , 125.37) | 1.2 (-30.8 , 71.2) | -20.3 (-26.0 , -15.2) | -19.3 (-46.9 , 37.1) |
| **Sex** |  |  |  |  |  |  |  |  |
| Males | 188.42 (120.30 , 267.31) | 37.3 (-7.4 , 133.3) | -15.3 (-21.3 , -10.2) | 16.3 (-23.3 , 101.1) | 97.39 (61.97 , 138.59) | 5.5 (-28.9 , 79.5) | -24.0 (-29.4 , -19.1) | -19.8 (-47.3 , 39.1) |
| Females | 177.86 (113.84 , 251.38) | 25.0 (-13.8 , 112.1) | -11.7 (-17.7 , -5.9) | 10.3 (-25.9 , 85.1) | 79.35 (50.31 , 113.40) | -4.5 (-33.8 , 61.2) | -16.0 (-22.4 , -10.1) | -19.9 (-46.9 , 34.1) |
| **Age class** |  |  |  |  |  |  |  |  |
| Under 5 | 1.48 (0.95 , 2.27) | -21.2 (-46.5 , 17.7) | -26.9 (-40.7 , -9.1) | -42.4 (-60.5 , -12.1) |  |  |  |  |
| 5-14 | 0.11 (0.05 , 0.21) | -41.0 (-67.2 , 22.8) | -29.6 (-42.1 , -16.6) | -58.4 (-76.5 , -16.2) |  |  |  |  |
| 15-49 | 41.31 (26.09 , 60.29) | -6.9 (-34.1 , 58.7) | 6.8 (-5.1 , 18.3) | -0.6 (-32.7 , 66.0) |  |  |  |  |
| 50-74 | 156.41 (97.09 , 226.26) | 16.8 (-21.0 , 96.9) | -26.0 (-31.4 , -21.1) | -13.6 (-43.6 , 47.1) |  |  |  |  |
| 75 plus | 373.36 (249.51 , 513.18) | 1.5 (-31.3 , 76.8) | -27.1 (-31.4 , -23.3) | -26.0 (-51.5 , 27.9) |  |  |  |  |
| **Cause** |  |  |  |  |  |  |  |  |
| Type 2 diabetes mellitus | 111.98 (62.77 , 174.44) | 95.0 (45.7 , 201.1) | -13.6 (-21.1 , -6.9) | 68.5 (19.7 , 159.5) | 54.77 (30.33 , 84.63) | 50.6 (12.8 , 133.7) | -18.7 (-26.2 , -12.1) | 22.5 (-13.0 , 89.9) |
| Chronic obstructive pulmonary disease | 31.96 (20.85 , 45.30) | -3.9 (-41.7 , 84.8) | -13.4 (-18.7 , -8.6) | -16.8 (-50.0 , 59.3) | 13.40 (8.76 , 19.18) | -29.9 (-57.4 , 34.4) | -25.4 (-30.0 , -21.1) | -47.7 (-68.7 , 0.4) |
| Stroke | 24.10 (16.18 , 33.04) | -25.8 (-51.1 , 34.4) | -14.6 (-19.5 , -10.2) | -36.7 (-58.9 , 12.8) | 12.69 (8.47 , 17.23) | -38.4 (-59.0 , 9.9) | -20.9 (-25.3 , -17.0) | -51.3 (-68.0 , -13.3) |
| Ischemic heart disease | 12.64 (7.78 , 18.90) | -10.4 (-38.4 , 54.0) | -9.6 (-14.6 , -5.1) | -19.1 (-45.8 , 40.7) | 5.69 (3.50 , 8.57) | -30.8 (-52.6 , 17.9) | -19.0 (-23.5 , -15.0) | -43.9 (-62.5 , -2.9) |
| Tracheal, bronchus, and lung cancer | 1.89 (1.11 , 2.83) | -2.7 (-35.2 , 69.7) | -19.8 (-35.0 , -3.9) | -21.9 (-51.4 , 39.3) | 0.85 (0.50 , 1.27) | -23.5 (-49.2 , 32.4) | -29.0 (-43.0 , -14.4) | -45.7 (-65.8 , -3.5) |
| Lower respiratory infections | 0.39 (0.20 , 0.72) | -9.9 (-46.6 , 78.5) | -20.4 (-25.9 , -15.1) | -28.3 (-57.9 , 40.1) | 0.25 (0.13 , 0.46) | -31.8 (-59.4 , 34.1) | -29.8 (-34.7 , -25.2) | -52.2 (-72.0 , -6.9) |
| Neonatal disorders | 0.01 (0.01 , 0.02) | -21.7 (-42.6 , 3.7) | -34.4 (-53.0 , -9.4) | -48.6 (-63.6 , -29.7) | 0.03 (0.02 , 0.05) | -17.3 (-39.4 , 9.4) | -17.4 (-40.8 , 14.2) | -31.7 (-51.6 , -6.5) |

UI: Uncertainty Interval

**Table S4.** Cause-specific overall burden of conditions that include ambient particulate matter and ozone pollution among their risk factors, in terms of crude and age-standardized rates (Italy, 2019) and temporal change (Italy, 1990–2019)

|  | **Crude rate per 100,000 inhabitants** | | | | **Age-standardized rate per 100,000 inhabitants** | | | |
| --- | --- | --- | --- | --- | --- | --- | --- | --- |
|  | **Estimate (95% UI)**  **for 2019** | **Percent change (95% UI)** | | | **Estimate (95% UI)**  **for 2019** | **Percent change (95% UI)** | | |
|  |  | **from 1990 to 2010** | **from 2010 to 2019** | **from 1990 to 2019** |  | **from 1990 to 2010** | **from 2010 to 2019** | **from 1990 to 2019** |
| **DALYs** |  |  |  |  |  |  |  |  |
| LRI | 230.98 (200.85 , 246.98) | -22.8 (-28.3 , -19.3) | 15.7 (11.8 , 19.5) | -10.7 (-19.0 , -5.4) | 98.65 (89.52 , 104.08) | -54.0 (-57.2 , -51.7) | -7.5 (-10.3 , -4.9) | -57.5 (-60.7 , -55.0) |
| COPD | 837.04 (741.68 , 917.58) | 1.4 (-4.7 , 7.7) | 9.7 (6.9 , 12.8) | 11.2 (3.6 , 18.7) | 324.98 (290.28 , 358.56) | -27.7 (-31.5 , -23.0) | -6.7 (-8.8 , -4.6) | -32.6 (-36.3 , -27.7) |
| TBL cancer | 1,158.93 (1,086.85 , 1,208.15) | -10.6 (-13.5 , -8.6) | -2.4 (-5.3 , 0.1) | -12.8 (-17.1 , -9.3) | 552.00 (523.27 , 573.25) | -28.3 (-30.1 , -26.8) | -12.8 (-15.2 , -10.5) | -37.4 (-40.0 , -35.1) |
| IHD | 2,148.99 (1,899.40 , 2,310.45) | -31.6 (-36.1 , -29.2) | 1.6 (-1.0 , 4.0) | -30.5 (-36.1 , -27.2) | 855.57 (778.85 , 910.48) | -50.7 (-53.0 , -49.3) | -14.0 (-15.9 , -12.2) | -57.6 (-59.8 , -56.0) |
| Stroke | 1,441.85 (1,278.83 , 1,557.79) | -35.2 (-39.6 , -32.4) | 3.8 (1.4 , 5.9) | -32.7 (-38.2 , -29.4) | 559.01 (504.58 , 599.16) | -54.3 (-56.8 , -52.5) | -12.2 (-14.0 , -10.5) | -59.9 (-62.4 , -58.2) |
| T2DM | 1,283.56 (1,021.92 , 1,580.28) | 36.4 (25.3 , 46.9) | 3.8 (-0.1 , 8.0) | 41.6 (29.6 , 52.5) | 586.64 (455.50 , 742.84) | 5.5 (-3.3 , 13.7) | -4.7 (-8.7 , -0.2) | 0.6 (-9.1 , 9.1) |
| Neonatal disorders | 222.98 (190.11 , 257.29) | -44.7 (-50.8 , -39.4) | -23.5 (-29.7 , -17.1) | -57.7 (-63.2 , -52.7) | 407.16 (351.53 , 461.59) | -49.2 (-55.5 , -44.4) | -18.1 (-25.9 , -9.4) | -58.4 (-65.2 , -52.7) |
| **Deaths** |  |  |  |  |  |  |  |  |
| LRI | 20.55 (17.15 , 22.37) | 4.8 (-4.0 , 9.8) | 29.1 (24.9 , 33.8) | 35.2 (21.6 , 43.6) | 6.56 (5.59 , 7.08) | -40.6 (-44.3 , -38.2) | -1.7 (-4.6 , 1.6) | -41.6 (-45.9 , -38.7) |
| COPD | 46.56 (39.26 , 51.15) | 16.1 (5.1 , 25.7) | 16.0 (11.4 , 21.1) | 34.7 (19.8 , 47.0) | 14.77 (12.63 , 16.23) | -27.1 (-32.9 , -21.4) | -7.8 (-10.8 , -4.2) | -32.8 (-38.9 , -27.1) |
| TBL cancer | 61.30 (56.24 , 64.47) | 7.1 (2.1 , 10.0) | 1.2 (-1.8 , 4.1) | 8.4 (1.1 , 13.3) | 25.57 (23.78 , 26.72) | -20.0 (-22.8 , -18.1) | -11.3 (-13.8 , -9.0) | -29.0 (-32.6 , -26.1) |
| IHD | 167.72 (141.62 , 184.97) | -15.8 (-23.1 , -12.0) | 9.2 (6.2 , 11.8) | -8.1 (-18.0 , -2.9) | 55.27 (47.69 , 60.39) | -46.8 (-50.6 , -44.8) | -13.8 (-15.6 , -11.9) | -54.1 (-57.6 , -52.1) |
| Stroke | 111.57 (93.53 , 123.69) | -22.5 (-29.8 , -18.3) | 10.5 (7.4 , 13.4) | -14.3 (-23.7 , -9.0) | 35.20 (30.05 , 38.56) | -52.1 (-55.9 , -49.9) | -13.4 (-15.5 , -11.3) | -58.6 (-62.2 , -56.4) |
| T2DM | 35.02 (29.92 , 37.68) | 2.8 (-5.4 , 8.5) | 10.7 (6.8 , 14.5) | 13.8 (2.6 , 21.4) | 11.92 (10.39 , 12.71) | -31.0 (-35.3 , -27.8) | -9.2 (-12.2 , -6.4) | -37.3 (-42.1 , -33.9) |
| Neonatal disorders | 1.00 (0.82 , 1.17) | -61.7 (-67.6 , -57.2) | -40.9 (-49.7 , -31.1) | -77.4 (-82.6 , -73.0) | 2.81 (2.30 , 3.29) | -59.6 (-65.8 , -54.8) | -25.6 (-36.6 , -13.3) | -69.9 (-76.9 , -64.1) |
| **YLLs** |  |  |  |  |  |  |  |  |
| LRI | 226.02 (196.30 , 241.98) | -23.4 (-29.1 , -19.9) | 16.0 (12.1 , 19.9) | -11.2 (-19.8 , -5.8) | 95.45 (86.31 , 100.83) | -54.7 (-57.8 , -52.4) | -7.5 (-10.2 , -4.8) | -58.1 (-61.3 , -55.6) |
| COPD | 530.22 (460.55 , 584.40) | -7.1 (-15.0 , 1.5) | 8.9 (5.0 , 13.5) | 1.2 (-9.0 , 11.1) | 192.04 (169.56 , 214.88) | -35.8 (-40.8 , -29.0) | -8.6 (-11.5 , -5.1) | -41.3 (-46.4 , -35.2) |
| TBL cancer | 1,142.24 (1,071.39 , 1,190.51) | -10.9 (-13.8 , -8.9) | -2.5 (-5.3 , 0.1) | -13.1 (-17.4 , -9.6) | 544.44 (516.52 , 565.54) | -28.5 (-30.3 , -27.1) | -12.8 (-15.3 , -10.5) | -37.6 (-40.2 , -35.3) |
| IHD | 2,030.36 (1,790.16 , 2,196.28) | -33.0 (-37.4 , -30.5) | 1.0 (-1.8 , 3.4) | -32.3 (-37.9 , -29.0) | 806.79 (732.37 , 858.97) | -51.7 (-54.0 , -50.3) | -14.8 (-16.7 , -12.9) | -58.8 (-61.2 , -57.2) |
| Stroke | 1,241.02 (1,080.45 , 1,343.58) | -38.1 (-42.8 , -35.2) | 2.8 (0.1 , 5.2) | -36.4 (-42.1 , -32.9) | 463.09 (415.02 , 492.89) | -57.2 (-59.9 , -55.6) | -14.2 (-16.2 , -12.3) | -63.3 (-65.9 , -61.7) |
| T2DM | 458.00 (405.36 , 484.93) | -15.7 (-20.8 , -11.9) | 4.4 (0.8 , 7.8) | -11.9 (-19.0 , -6.8) | 180.93 (163.68 , 190.60) | -37.8 (-40.8 , -35.3) | -10.0 (-13.2 , -7.0) | -44.0 (-47.4 , -41.1) |
| Neonatal disorders | 89.01 (72.73 , 104.19) | -61.7 (-67.6 , -57.2) | -40.9 (-49.7 , -31.1) | -77.4 (-82.6 , -73.0) | 249.62 (203.94 , 292.27) | -59.6 (-65.8 , -54.8) | -25.6 (-36.6 , -13.3) | -69.9 (-76.9 , -64.1) |
| **YLDs** |  |  |  |  |  |  |  |  |
| LRI | 4.96 (3.38 , 7.02) | 15.5 (10.0 , 21.7) | 2.6 (-1.7 , 7.3) | 18.5 (12.2 , 24.5) | 3.20 (2.16 , 4.57) | -13.9 (-18.5 , -9.0) | -9.4 (-13.3 , -5.4) | -22.0 (-25.8 , -18.2) |
| COPD | 306.82 (241.66 , 366.41) | 20.7 (15.6 , 25.4) | 11.2 (8.2 , 14.0) | 34.2 (26.4 , 40.9) | 132.94 (105.44 , 159.29) | -10.7 (-13.7 , -7.8) | -3.8 (-6.0 , -1.3) | -14.1 (-17.7 , -10.7) |
| TBL cancer | 16.70 (11.67 , 22.43) | 19.7 (12.0 , 27.6) | 0.9 (-16.9 , 20.7) | 20.8 (-1.6 , 45.1) | 7.57 (5.24 , 10.21) | -5.8 (-11.7 , 0.7) | -10.6 (-27.1 , 7.6) | -15.7 (-31.2 , 2.0) |
| IHD | 118.63 (79.73 , 166.93) | 12.9 (4.5 , 23.5) | 14.3 (10.6 , 18.3) | 29.1 (18.3 , 42.7) | 48.78 (33.11 , 68.16) | -17.0 (-22.2 , -10.4) | 0.4 (-2.6 , 3.4) | -16.7 (-21.7 , -10.3) |
| Stroke | 200.83 (144.76 , 257.96) | -6.2 (-10.6 , -1.4) | 10.5 (7.9 , 13.6) | 3.7 (-1.7 , 10.0) | 95.92 (68.80 , 122.02) | -25.5 (-28.5 , -22.3) | -1.0 (-2.9 , 1.3) | -26.3 (-29.5 , -23.0) |
| T2DM | 825.56 (566.93 , 1,131.26) | 106.5 (91.9 , 122.7) | 3.5 (-1.9 , 9.5) | 113.7 (103.5 , 126.0) | 405.71 (275.72 , 559.65) | 59.3 (48.5 , 71.6) | -2.2 (-8.0 , 4.3) | 55.9 (48.6 , 64.2) |
| Neonatal disorders | 133.97 (104.88 , 165.01) | 5.3 (-2.9 , 14.5) | -4.8 (-13.0 , 4.1) | 0.2 (-10.8 , 11.9) | 157.54 (123.08 , 194.75) | 9.1 (0.5 , 18.7) | -2.7 (-11.1 , 6.4) | 6.2 (-5.6 , 18.3) |

UI: Uncertainty Interval, LRI: Lower respiratory infections, COPD: Chronic obstructive pulmonary disease, TBL: Tracheal, bronchus and lung, IHD: Ischemic heart disease, T2DM: Type 2 diabetes mellitus

**Figure S1.** Yearly estimated population-weighted average exposure to particulate matter with diameter less than 2.5μm (PM_2.5_) (A) and ozone (B), for the years available within the Global Burden of Disease Study 2019 (Italy, 1990–2019)


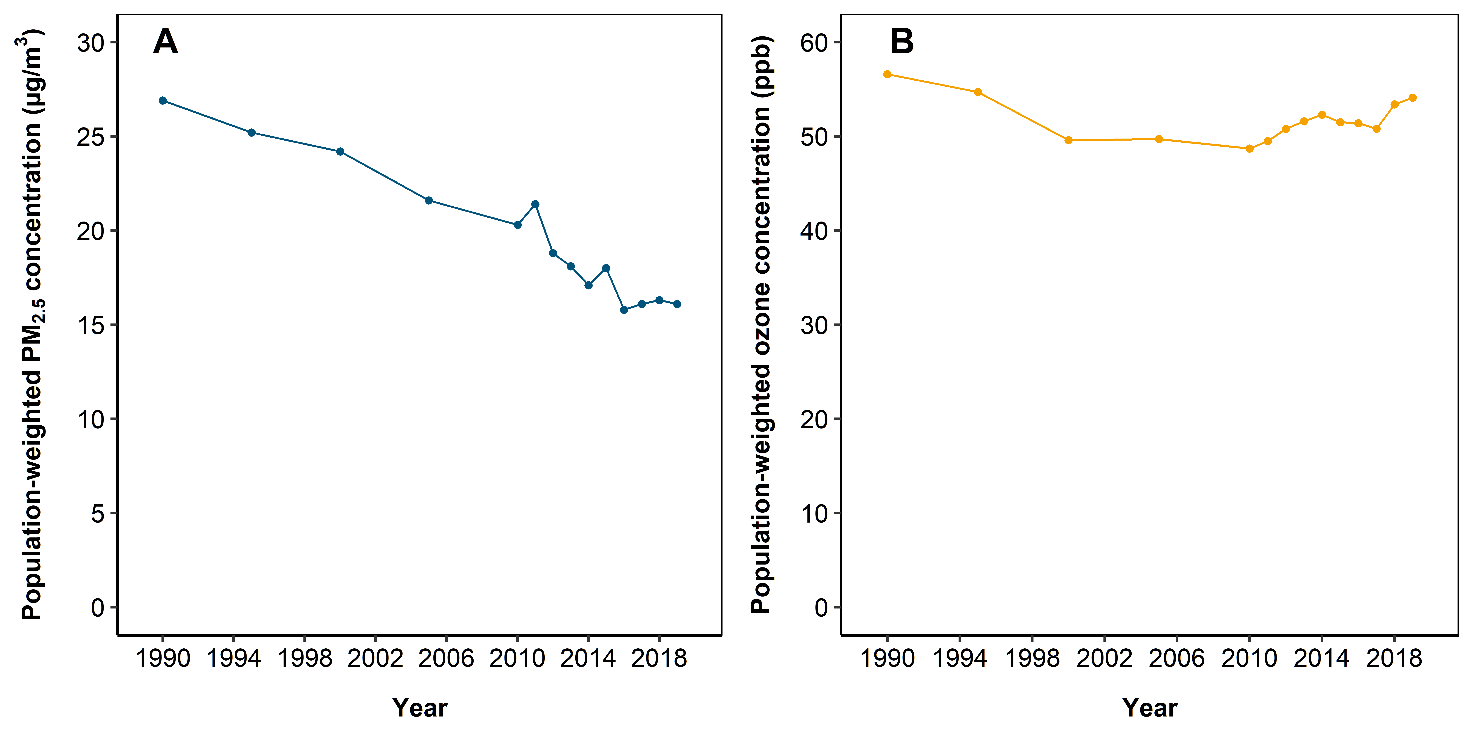


**Figure S2.** Lower respiratory infections. Time-series of the estimated age-standardized rates (per 100,000 inhabitants) of disability adjusted life years (DALYs) (A), mortality (B), years lived in disability (YLDs) (C), and years of life lost (YLLs) (D). Comparison between overall rates and rates due to ambient particulate matter pollution (Italy, 1990–2019). Whiskers represent 95% Uncertainty Intervals.


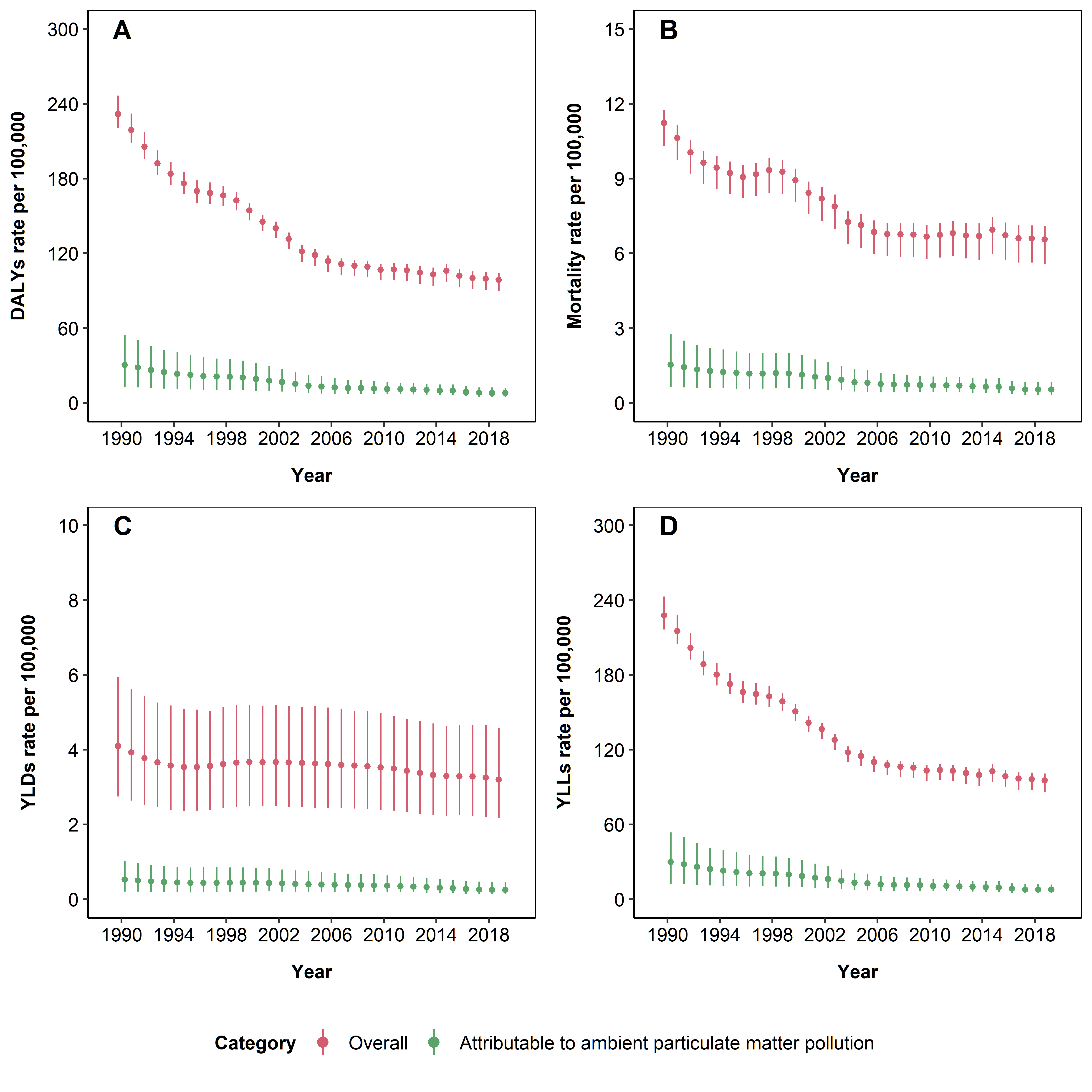


**Figure S3.** Chronic obstructive pulmonary disease. Time-series of the estimated age-standardized rates (per 100,000 inhabitants) of disability adjusted life years (DALYs) (A), mortality (B), years lived in disability (YLDs) (C), and years of life lost (YLLs) (D). Comparison between overall rates and rates due to ambient particulate matter and ozone pollution (Italy, 1990–2019). Whiskers represent 95% Uncertainty Intervals.


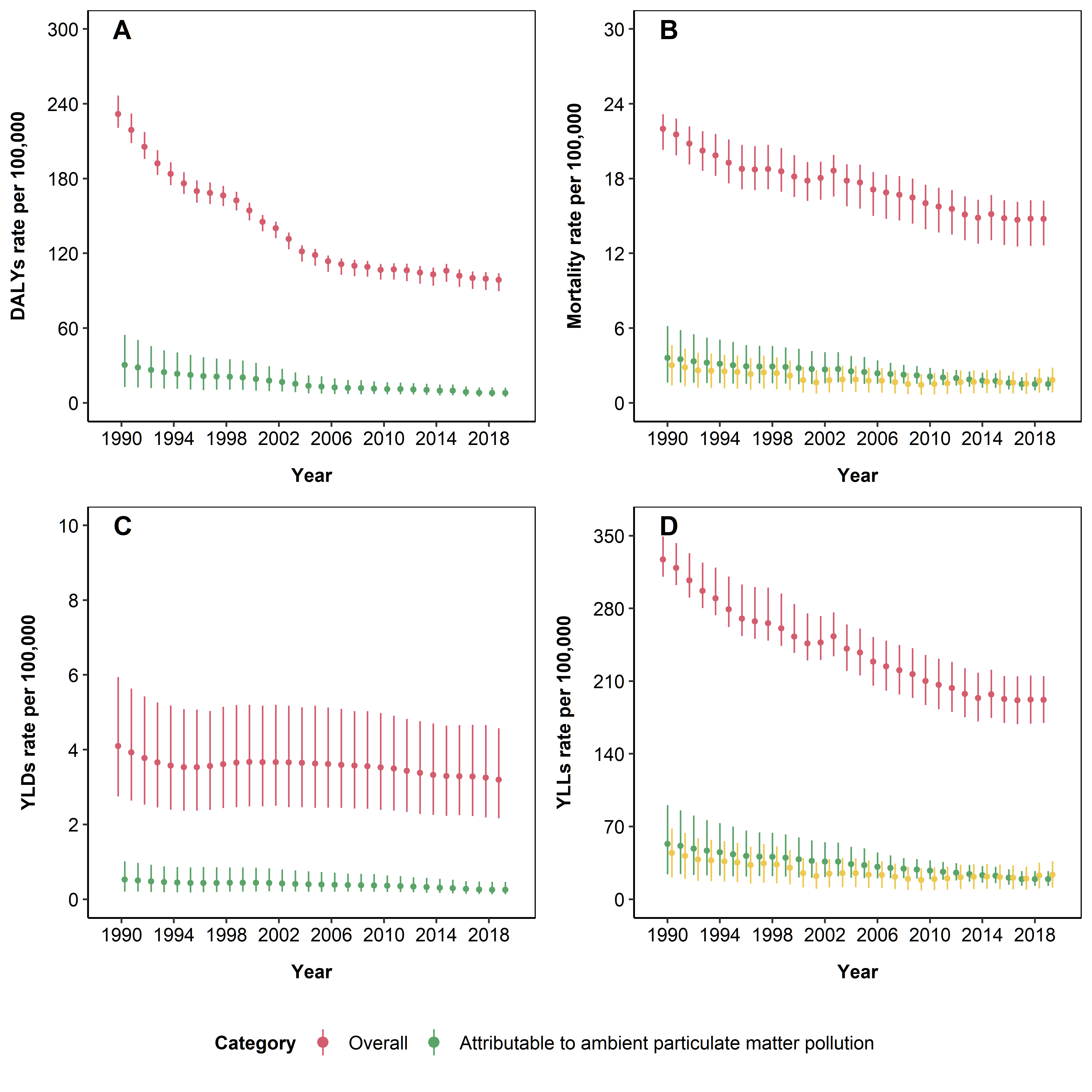


**Figure S4.** Tracheal, bronchus, and lung cancer. Time-series of the estimated age-standardized rates (per 100,000 inhabitants) of disability adjusted life years (DALYs) (A), mortality (B), years lived in disability (YLDs) (C), and years of life lost (YLLs) (D). Comparison between overall rates and rates due to ambient particulate matter pollution (Italy, 1990–2019). Whiskers represent 95% Uncertainty Intervals.


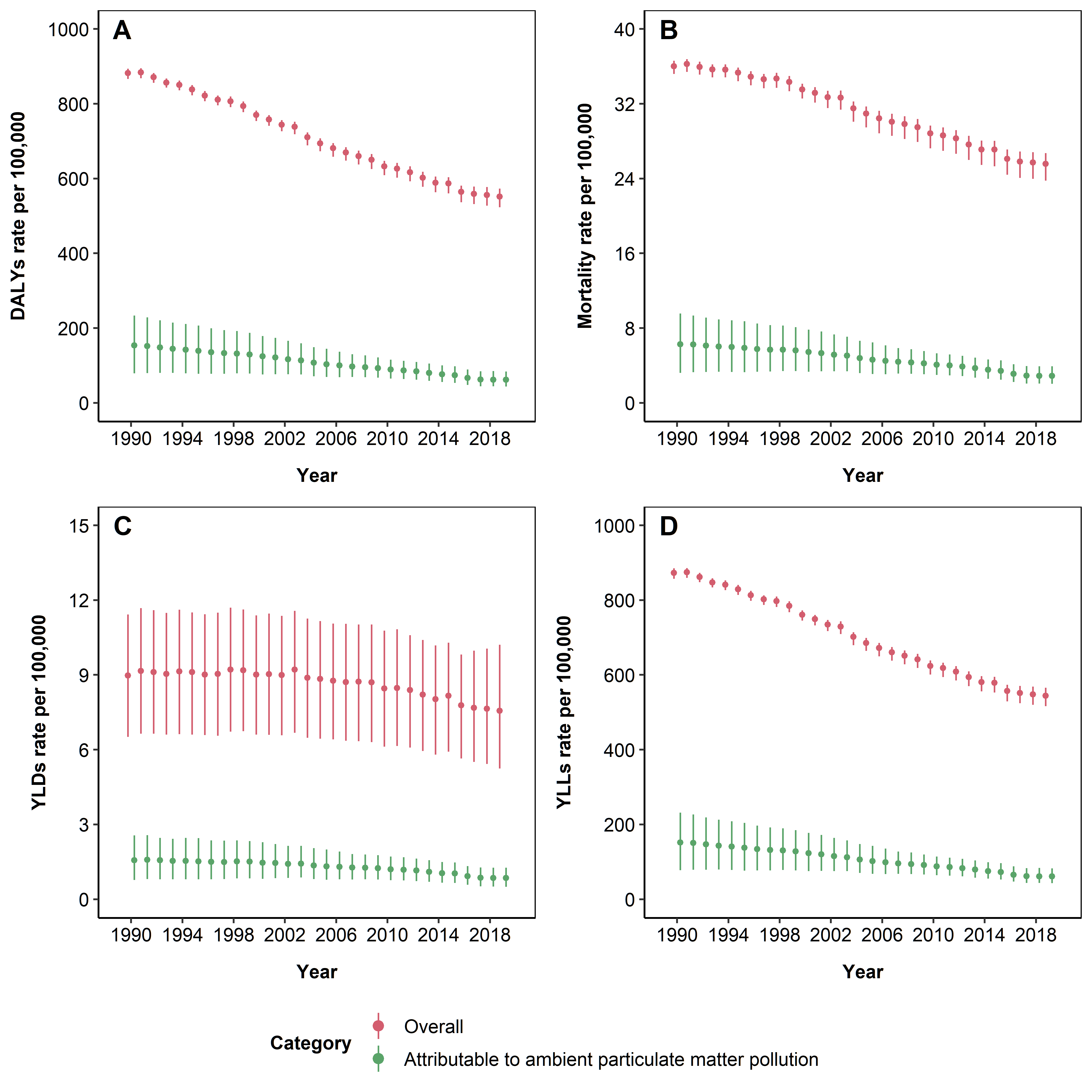


**Figure S5.** Ischemic heart disease. Time-series of the estimated age-standardized rates (per 100,000 inhabitants) of disability adjusted life years (DALYs) (A), mortality (B), years lived in disability (YLDs) (C), and years of life lost (YLLs) (D). Comparison between overall rates and rates due to ambient particulate matter pollution (Italy, 1990–2019). Whiskers represent 95% Uncertainty Intervals.


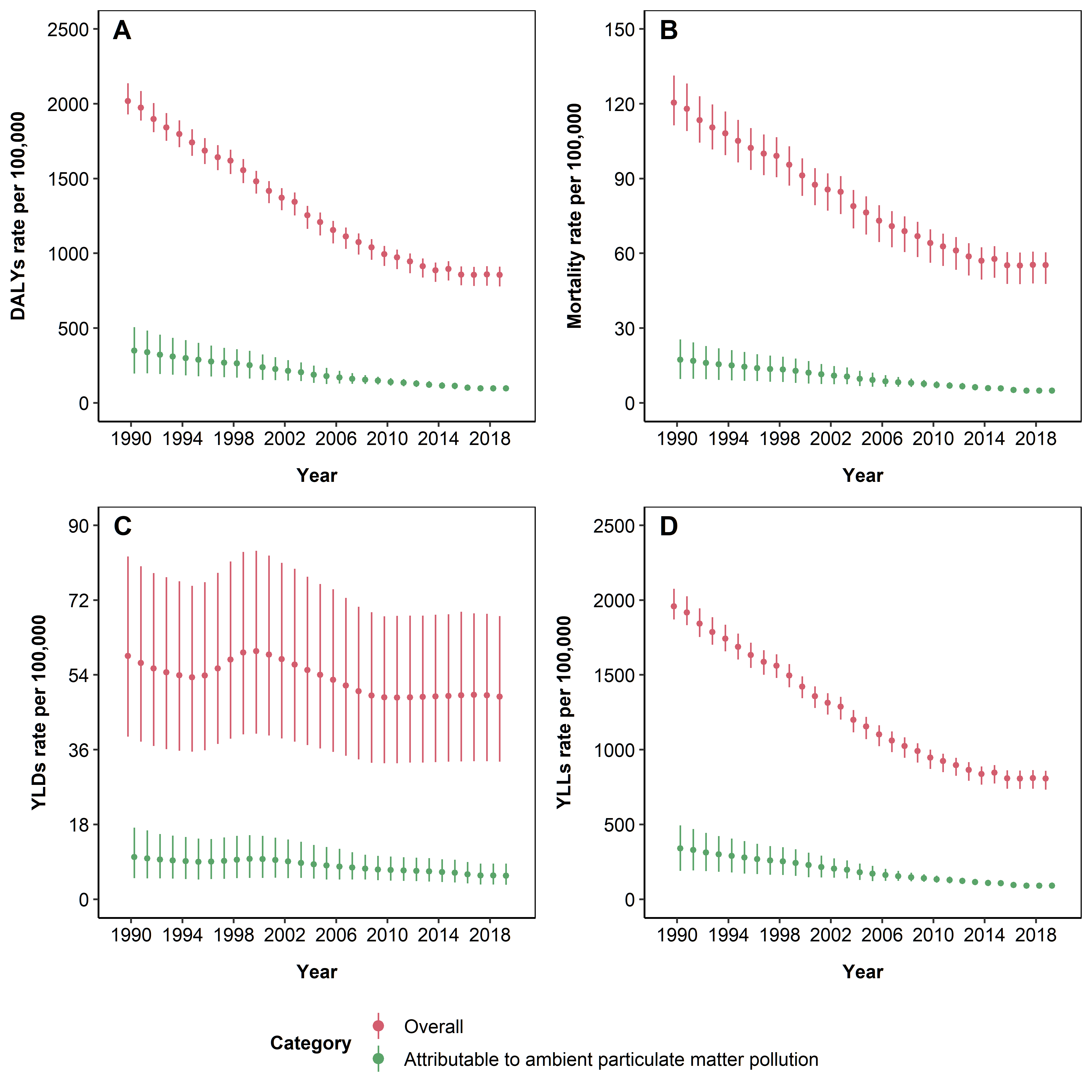


**Figure S6.** Stroke. Time-series of the estimated age-standardized rates (per 100,000 inhabitants) of disability adjusted life years (DALYs) (A), mortality (B), years lived in disability (YLDs) (C), and years of life lost (YLLs) (D). Comparison between overall rates and rates due to ambient particulate matter pollution (Italy, 1990–2019). Whiskers represent 95% Uncertainty Intervals.


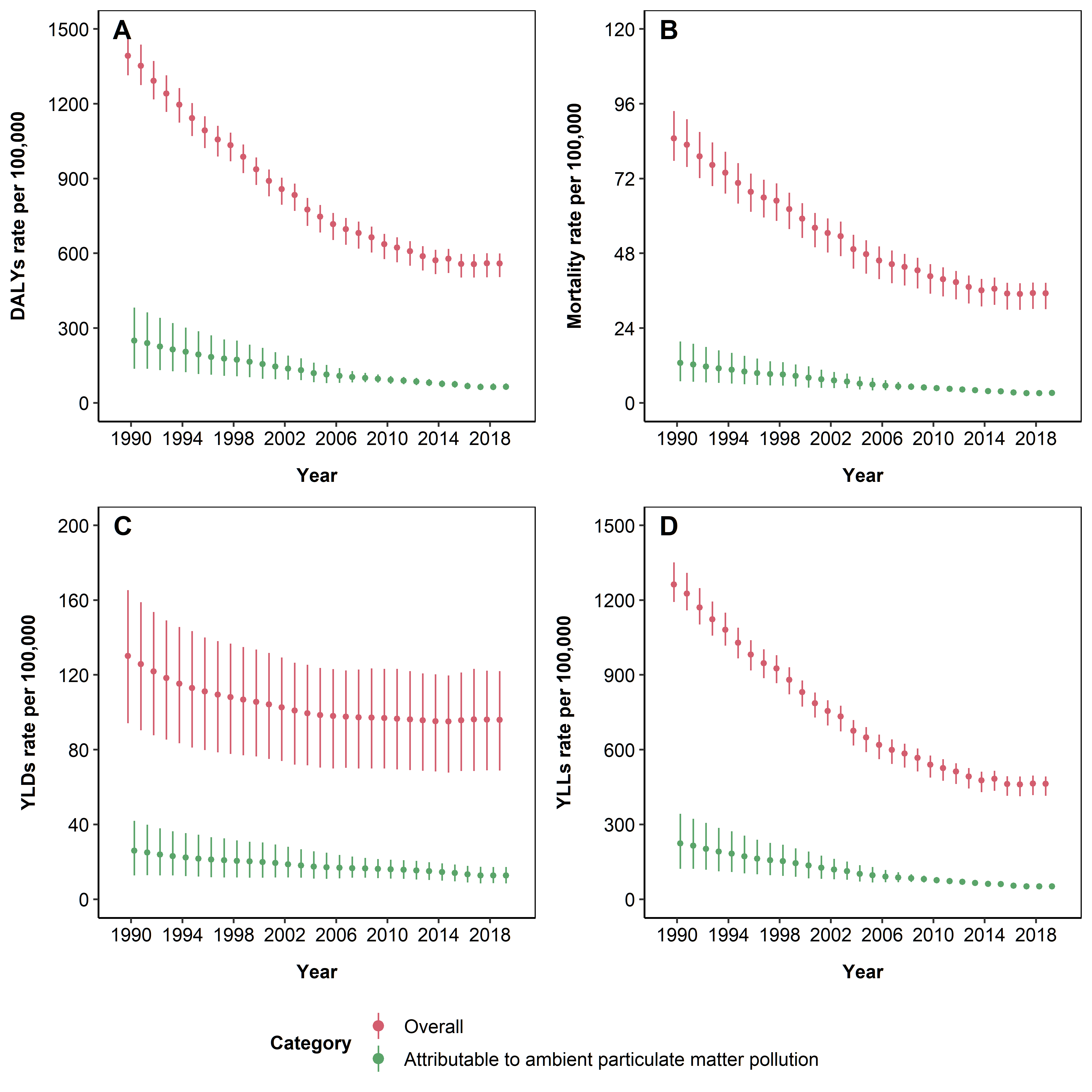


**Figure S7.** Type 2 diabetes mellitus. Time-series of the estimated age-standardized rates (per 100,000 inhabitants) of disability adjusted life years (DALYs) (A), mortality (B), years lived in disability (YLDs) (C), and years of life lost (YLLs) (D). Comparison between overall rates and rates due to ambient particulate matter pollution (Italy, 1990–2019). Whiskers represent 95% Uncertainty Intervals.


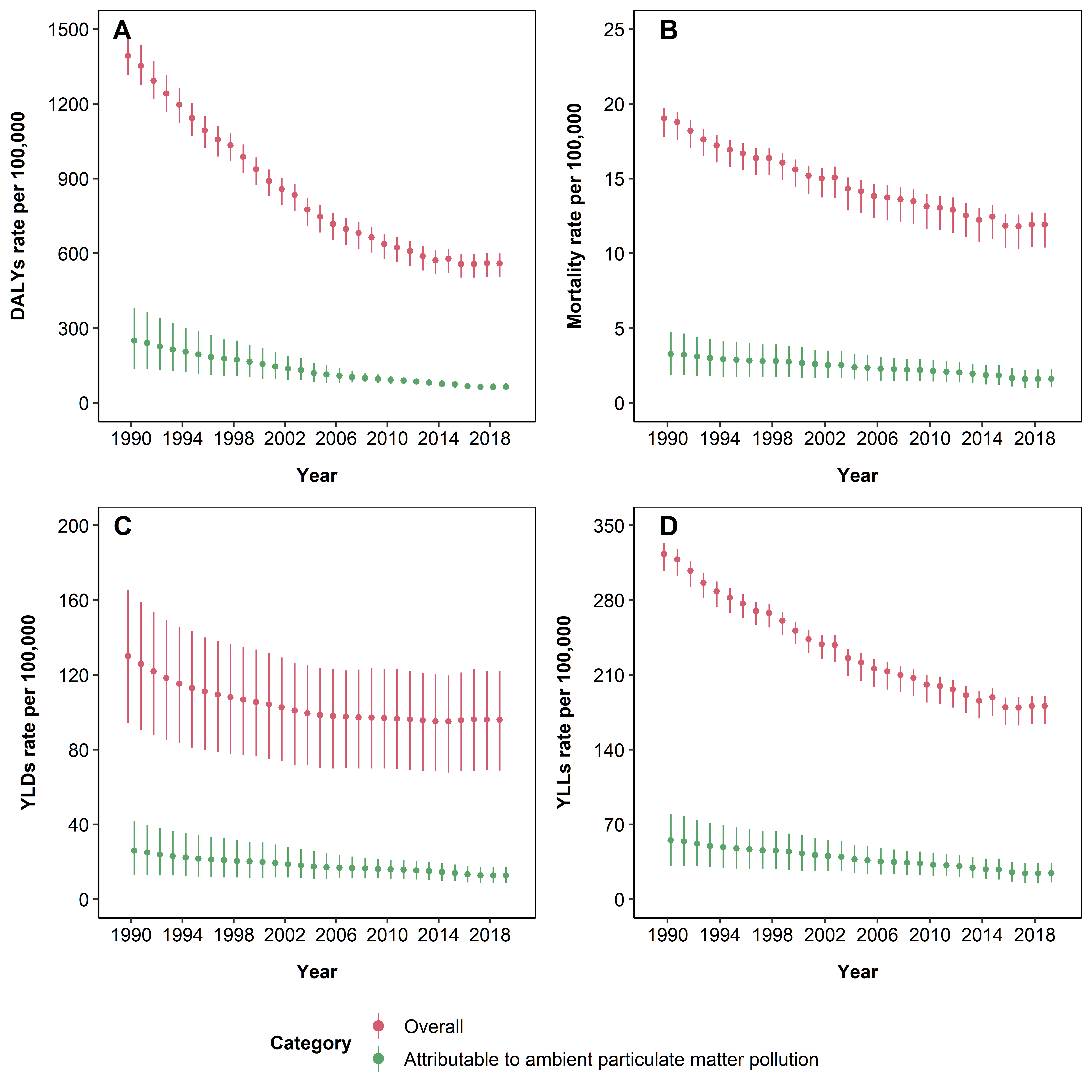


**Figure S8.** Neonatal disorders. Time-series of the estimated age-standardized rates (per 100,000 inhabitants) of mortality (A), disability adjusted life years (DALYs) (B), years of life lost (YLLs) (C), years lived in disability (YLDs) (D). Comparison between overall rates and rates due to ambient particulate matter pollution (Italy, 1990–2019). Whiskers represent 95% Uncertainty Intervals.


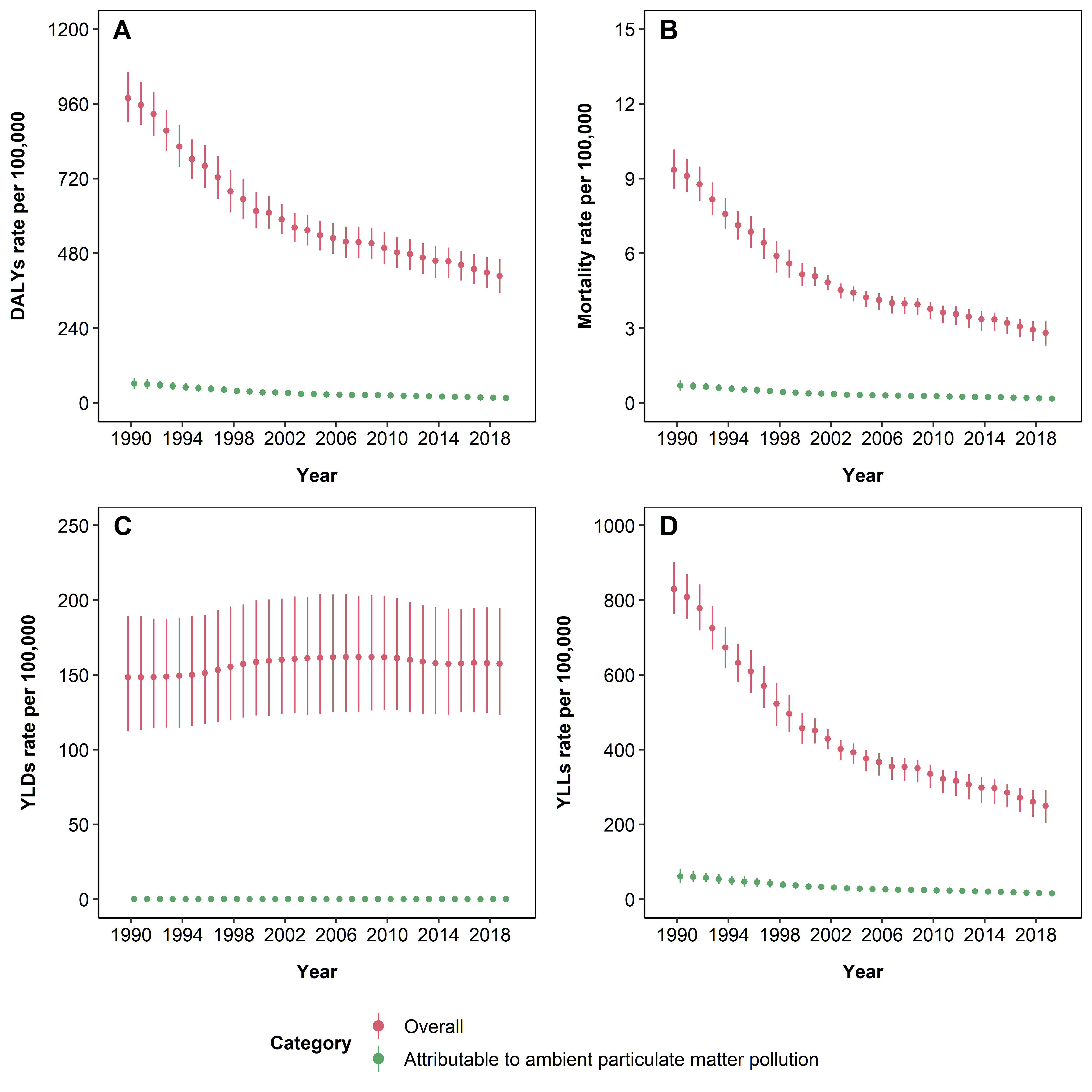


**Figure S9.** Yearly estimated age structure of the Italian population from 1990 to 2019 (A), and of the world population in 2019 (B), obtained from the Global Burden of Disease Study 2019


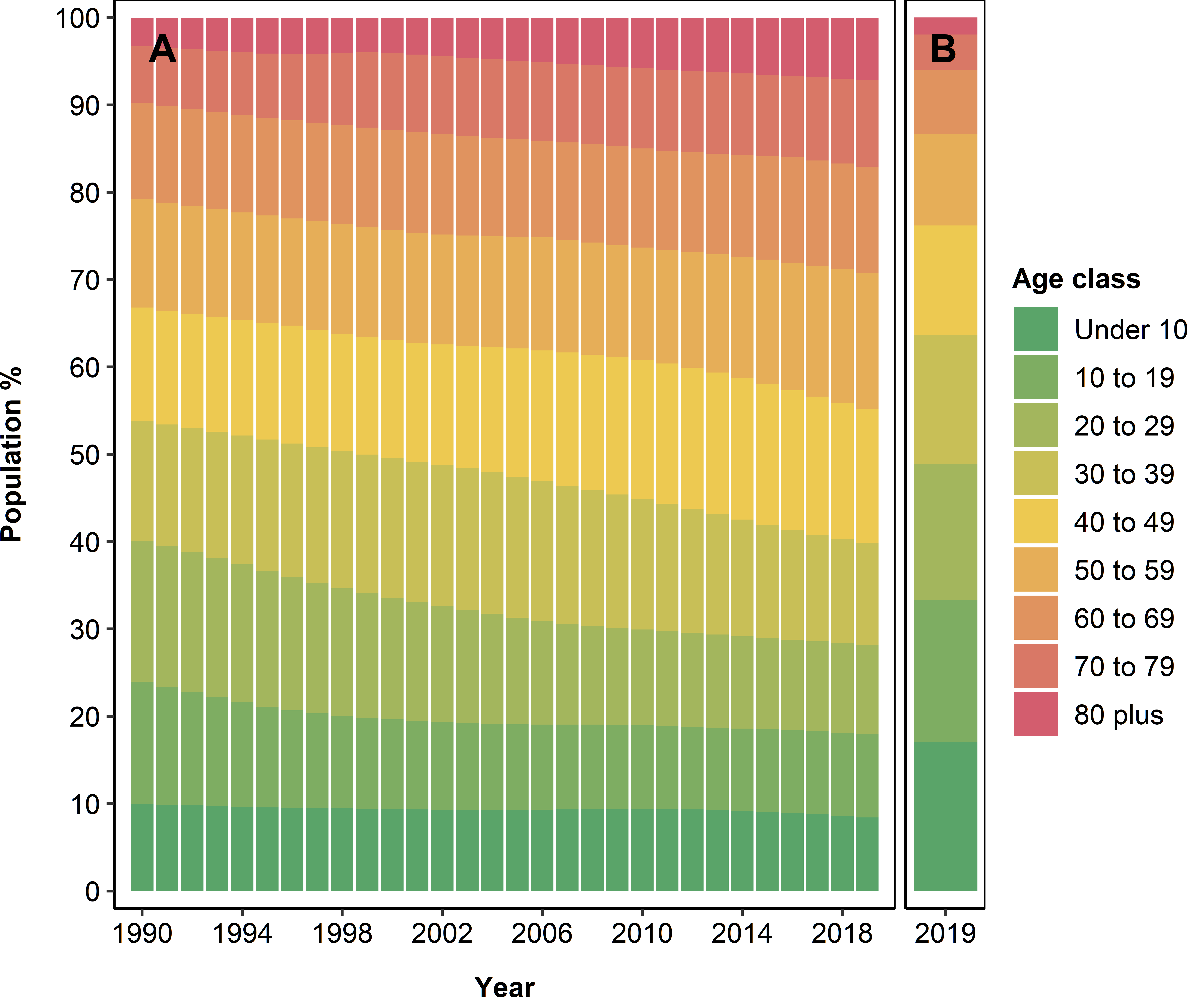

Supplement: Supplementary file 1 [file DataSheet1.docx]
